# Increased Expression of the RBPMS Splice Variants Inhibits Cell Proliferation in Ovarian Cancer Cells

**DOI:** 10.3390/ijms232314742

**Published:** 2022-11-25

**Authors:** Robert J. Rabelo-Fernández, Ricardo A. Noriega Rivera, Yasmarie Santana Rivera, José Tous-Beveraggi, Fatima Valiyeva, Pablo E. Vivas-Mejia

**Affiliations:** 1 Department of Biology, University of Puerto Rico at Rio Piedras, San Juan, PR 00925, USA; 2 Molecular Signaling and Experimental Therapy Laboratory, Comprehensive Cancer Center, University of Puerto Rico, San Juan, PR 00936, USA; 3 Department of Biochemistry, University of Puerto Rico, Medical Science Campus, San Juan, PR 00925, USA; 4 School of Dentistry, University of Puerto Rico, Medical Sciences Campus, San Juan, PR 00921, USA; 5 Departments of Interdisciplinary Sciences, University of Puerto Rico at Rio Piedras, San Juan, PR 00925, USA

**Keywords:** RNA Binding Protein with Multiple Splicing, cisplatin resistance, RBPMS variants, ovarian cancer, RBPMS

## Abstract

RNA-Binding Protein with Multiple Splicing (RBPMS) is a member of family proteins that bind to nascent RNA transcripts and regulate their splicing, localization, and stability. Evidence indicates that RBPMS controls the activity of transcription factors associated with cell growth and proliferation, including AP-1 and Smads. Three major RBPMS protein splice variants (RBPMSA, RBPMSB, and RBPMSC) have been described in the literature. We previously reported that reduced RBPMS levels decreased the sensitivity of ovarian cancer cells to cisplatin treatment. However, little is known about the biological role of the RBPMS splice variants in ovarian cancer cells. We performed RT-PCR and Western blots and observed that both RBPMSA and RBPMSC are reduced at the mRNA and protein levels in cisplatin resistant as compared with cisplatin sensitive ovarian cancer cells. The mRNA and protein levels of RBPMSB were not detectable in any of the ovarian cancer cells tested. To better understand the biological role of each RBPMSA and RBPMSC, we transfected these two splice variants in the A2780CP20 and OVCAR3CIS cisplatin resistant ovarian cancer cells and performed cell proliferation, cell migration, and invasion assays. Compared with control clones, a significant reduction in the number of colonies, colony size, cell migration, and invasion was observed with RBPMSA and RBPMSC overexpressed cells. Moreover, A2780CP20-RBPMSA and A2780CP20-RBPMSC clones showed reduced senescence-associated β-galactosidase (β-Gal)-levels when compared with control clones. A2780CP20-RBPMSA clones were more sensitive to cisplatin treatment as compared with A2780CP20-RBPMSC clones. The A2780CP20-RBPMSA and A2780CP20-RBPMSC clones subcutaneously injected into athymic nude mice formed smaller tumors as compared with A2780CP20-EV control group. Additionally, immunohistochemical analysis showed lower proliferation (Ki67) and angiogenesis (CD31) staining in tissue sections of A2780CP20-RBPMSA and A2780CP20-RBPMSC tumors compared with controls. RNAseq studies revealed many common RNA transcripts altered in A2780CP20-RBPMSA and A2780CP20-RBPMSC clones. Unique RNA transcripts deregulated by each RBPMS variant were also observed. Kaplan–Meier (KM) plotter database information identified clinically relevant RBPMSA and RBPMSC downstream effectors. These studies suggest that increased levels of RBPMSA and RBPMSC reduce cell proliferation in ovarian cancer cells. However, only RBPMSA expression levels were associated with the sensitivity of ovarian cancer cells to cisplatin treatment.

## 1. Introduction

Ovarian cancer is the most lethal gynecologic malignancy with an estimated 19,880 new cases and 12,810 deaths expected for 2022 in the United State [1]. Its high death rate is reflective of the fact that most ovarian cancer patients are diagnosed with advanced stages of the disease. Ovarian cancer ranks fifth in cancer deaths among women and is the most common female reproductive system malignancy in western countries. The survival rate of ovarian cancer is approximately 45% after five years of diagnosis. Depending on the cell origin, ovarian cancer is divided into four types: germ cells, sex-cord stromal, border cells, and epithelial cells. Epithelial ovarian tumors account for ~90% of ovarian cancers and can be subdivided into five major histological subtypes, which include high-grade serous, low-grade serous, endometrioid, clear-cell, and mucinous carcinomas [2,3,4]. The high-grade serous tumors (HGSOC) subtype is the most diagnosed [5,6]. The standard of care for women with ovarian cancer includes cytoreductive surgery and platinum/taxane-based combination chemotherapy. Unfortunately, despite initial response, relapse occurs in over 60% of patients, resulting in chemo-resistant fatal disease [7].

Decreased levels of the channels that reduce the influx of cisplatin inside the cells, increased levels of proteins or channels that promote cisplatin efflux, increased intracellular levels of specific sulfur containing macromolecules that reduce the nuclear cisplatin concentration, the deregulation of DNA repair mechanisms, and the metabolic rewiring could contribute to the cisplatin resistance phenotype of ovarian cancer cells [8]. Additionally, dysregulation of oncogenes [9], tumor suppressor genes [10], and non-coding RNAs (ncRNAs) promote cell survival pathways that contribute to cisplatin resistance [11]. However, the key molecules governing cisplatin resistance have not been fully elucidated.

RBPMS, known as HERMES (Heart RNA Recognition Motif Expressed Sequence), is a member of the RNA-binding proteins family located in chromosome 8p12 [12]. The RBPMS gene spans over 230 kb (30,241,924 to 30,430,508 bp) in the human genome [13] RBPMS’ alternative splicing renders multiple transcript variants encoding at least three different protein isoforms, which are designated as RBPMSA (NM_001008710), RBPMSB (NM_001008711), and RBPMSC (NM_001008712). The canonical protein is the RBPMSA variant which is commonly referred to as RBPMS (RBPMS1) [14,15]. Evidence indicates that RBPMS binds to the nascent RNA transcripts and regulates their processing, including the pre-mRNA splicing and the transport, localization, and stability of the RNA molecule [16]. RBPMS is also thought to bind to transcription factors to regulate gene expression [17]. For example, reports have shown that RBPMS interacts with Smad2, Smad3, and Smad4, promoting Smad-mediated transcriptional activity signaling pathways linked to cell growth, proliferation, and cell survival in vitro and in vivo [18,19]. Other reports indicate that RBPMS binds to c-Fos to eliminate the formation of the c-Fos/c-Jun or Smad3/c-Jun complexes [19]. In cultured cells and mouse xenograft models, RBPMS inhibited the growth and migration of breast cancer cells through its interaction with c-Fos or Smad3 [19]. Recently we published that reduced RBPMS levels increase the sensitivity of ovarian cancer cells to cisplatin treatment [17]. However, the role of each RBPMS splice variant in ovarian cancer cells has not been previously studied.

In the present study, we investigated the role of the RBPMS splice variants in ovarian cancer cells and mouse models. First, we assessed the protein and RNA levels of RBPMSA, RBPMSB, and RBPMSC in a panel of cisplatin sensitive and cisplatin resistant cells. Then, we investigated the biological effects of overexpressing RBPMSA or RBPMSC in the cisplatin resistant ovarian cancer cells, A2780CP20 and OVCAR3CIS. RNAseq studies identified common and specific RBPMA and RBPMSC downstream effectors. Interrogation of the KM plotter database (https://kmplot.com, accessed on 21 January 2021) revealed that some RBPMS downstream effectors correlate well with the overall survival (OS) and progression-free survival (PFS) of the disease. Overall, our studies suggest that reduced levels of RBPMSA and RBPMSC contribute to the cell growth, migration, and invasion ability of ovarian cancer cells. Additionally, increased levels of RBPMSA sensitize ovarian cancer cells to cisplatin treatment.

## 2. Results

### 2.1. RBPMSA and RBPMSC Protein Levels Are Reduced in Cisplatin Resistance Ovarian Cancer Cell Lines

**Figure 1:**
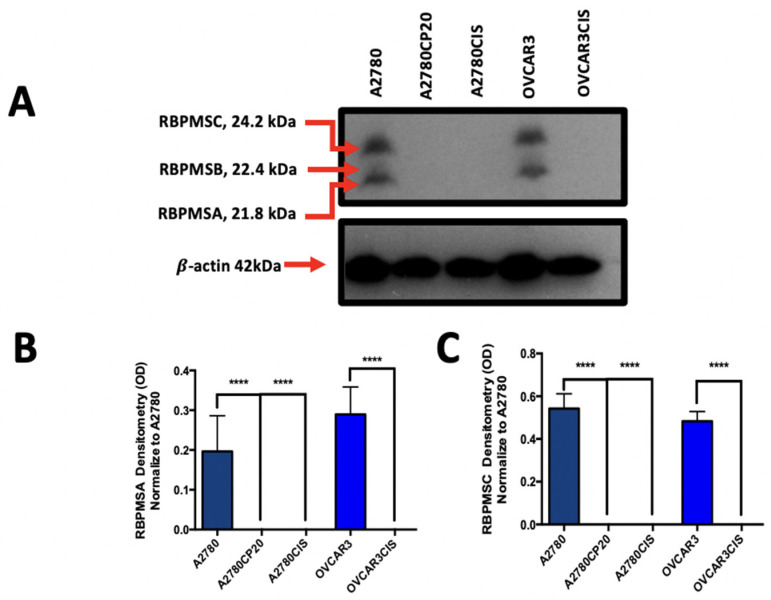

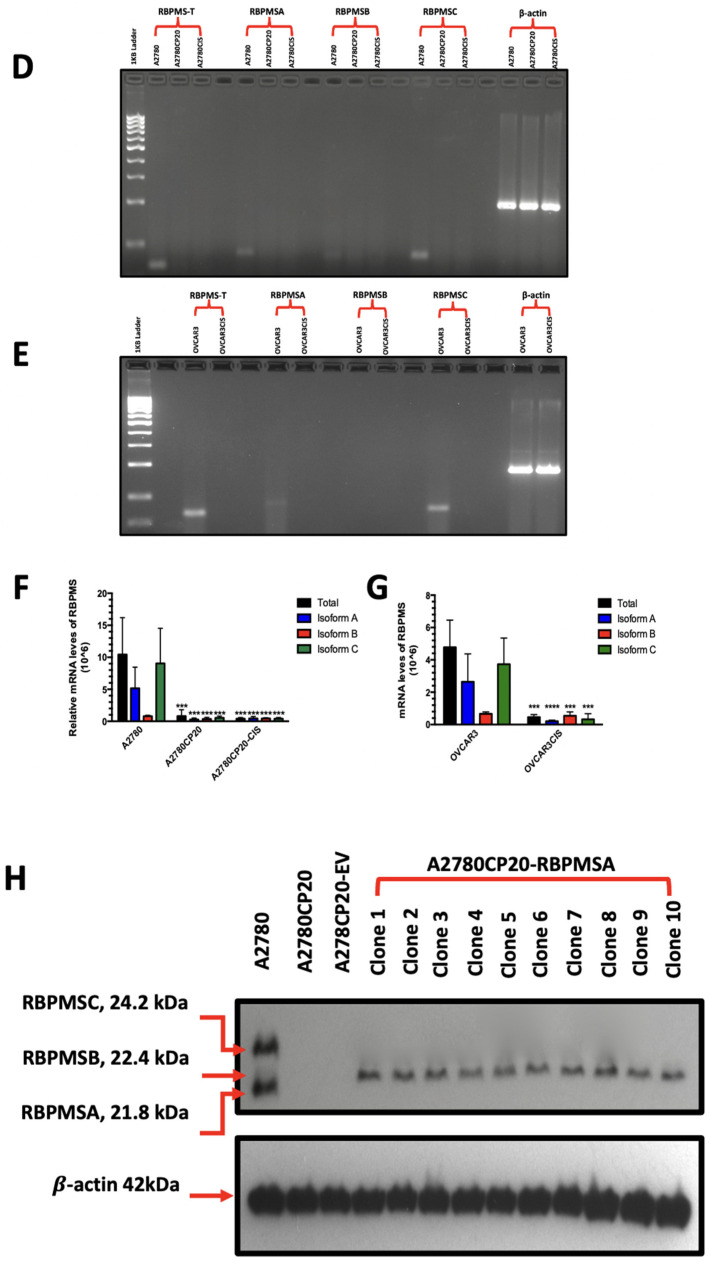

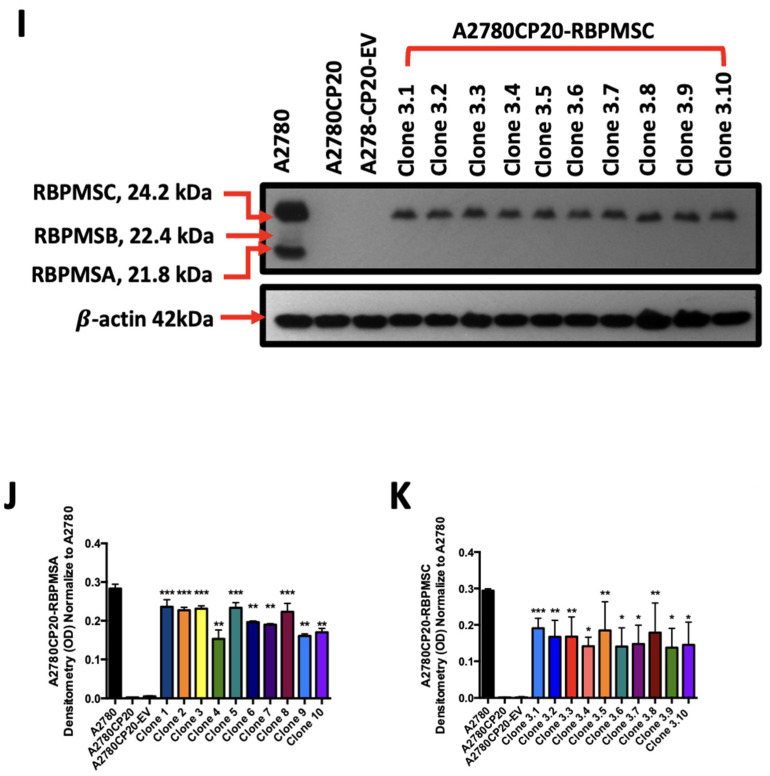
Protein and mRNA levels of RBPMS splice variants ovarian cancer cell lines and stable transfected clones. (**A**) Western blot analysis was performed with 50 µg of protein extracts, and β-actin was used as a loading control. (**B**,**C**) Densitometry analysis of band intensities shown in (**A**). (**D**,**E**) RT-PCR was performed starting with 100 μg of total RNA. DNA products were separated in 2% agarose gel electrophoresis and the gel was stained with Ethidium bromide. (**F**,**G**) Densitometry analysis of band intensities shown in (**D**,**E**). Fold changes at the protein and mRNA levels were calculated relative to the cisplatin sensitive cell pairs. Bars: averages ± SEM of three independent experiments. (**H**,**I**) Western Blot images obtained with 50 µg of proteins extracted from RBPMS and RBPMSC overexpression clones. (**J**,**K**) Densitometry analysis of band intensities, shown in (**H**,**I**). Fold changes in protein levels were calculated relative to the A2780CP20-EV clones. * *p* < 0.05, ** *p* < 0.01, *** *p* < 0.001, **** *p* < 0.0001.

To assesses the protein and mRNA levels of RBPMS splice variants, we performed Western blots and real-time PCR. The protein levels of RBPMSA and RBPMC levels were negligible in the cisplatin-resistant ovarian cancer cell lines (A2780CP20, A2780CIS, and OVCAR3CIS) when compared with their cisplatin-sensitive counterparts (A2780 and OVCAR3) (Figure 1A) (Original Western Blot images were included in Supplementary Figure S1). The half maximal inhibitory concentration (IC50) values of these cells to cisplatin have been published [20]. Densitometric analysis of the band intensities confirmed our observation (Figure 1B,C). RT-PCR results showed that mRNA levels of RBPMSA and RBPMSC were also significantly lower in cisplatin resistant as compared with cisplatin sensitive ovarian cancer cells (Figure 1D,E). Additionally, densitometric analysis of the PCR bands in the agarose gels confirmed the findings (Figure 1F,G). These results indicate that RBPMSA and RBPMSC levels in cisplatin-resistant ovarian cancer cells are reduced not only at protein level but also at the transcriptional level. RBPMSB splice variant were not detected in cisplatin sensitive ovarian cancer cell lines at the mRNA and protein levels. Therefore, we focus our studies on the RBPMSA and RBPMSC splice variants.

### 2.2. Overexpression of RBPMSA and RBPMSC Decreased Cell Growth and Proliferation of Cisplatin Resistant Ovarian Cancer Cells

**Figure 2:**
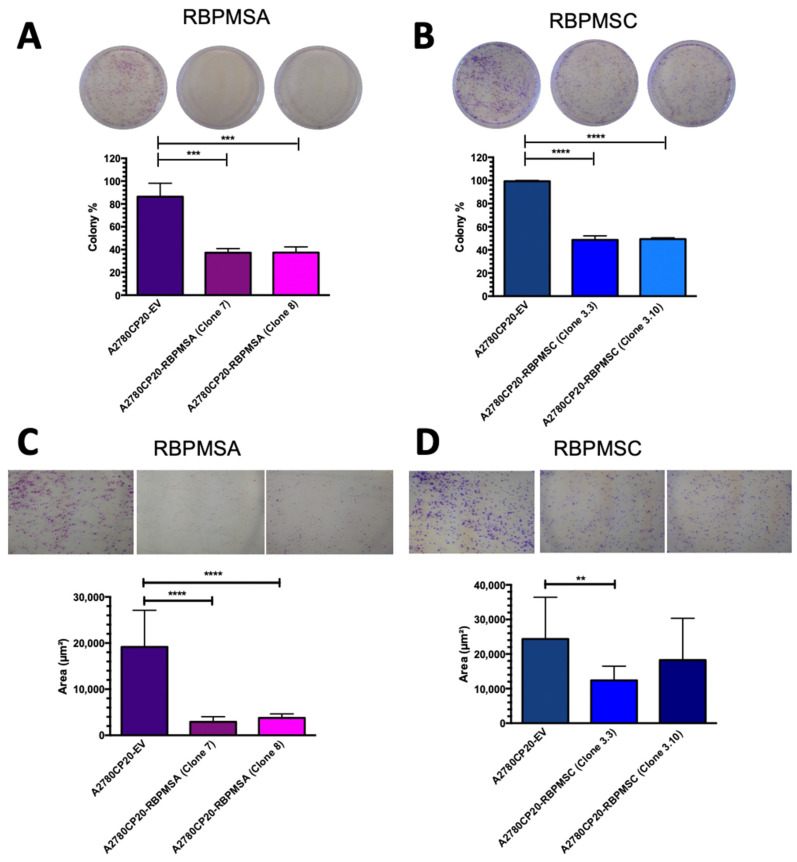

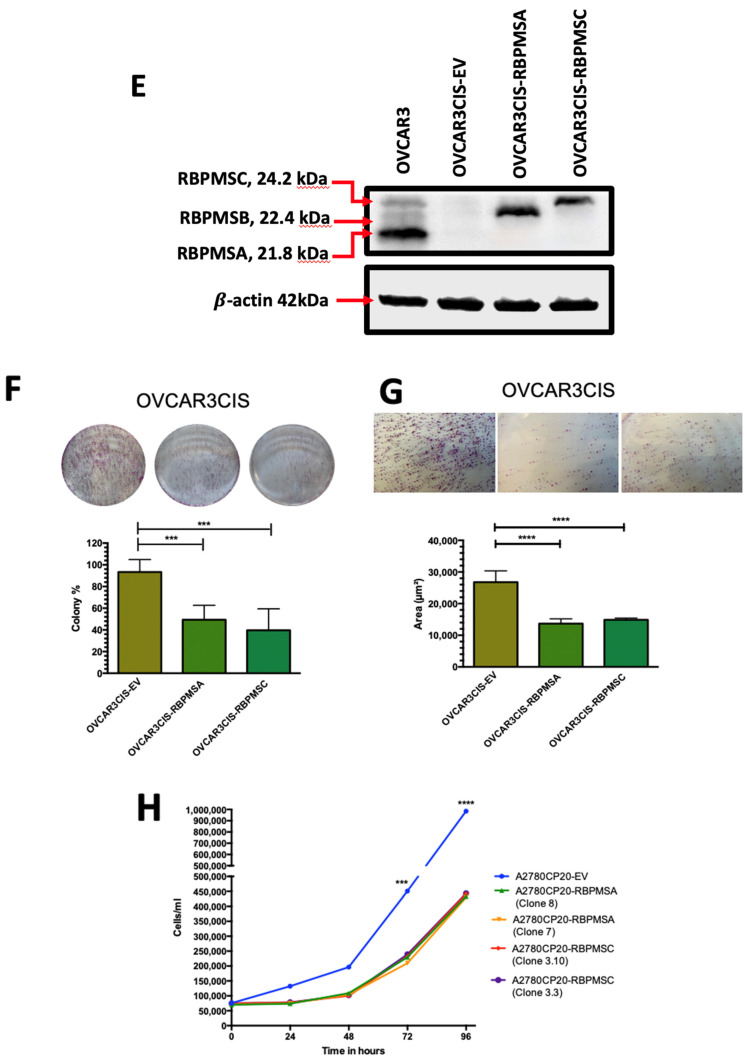

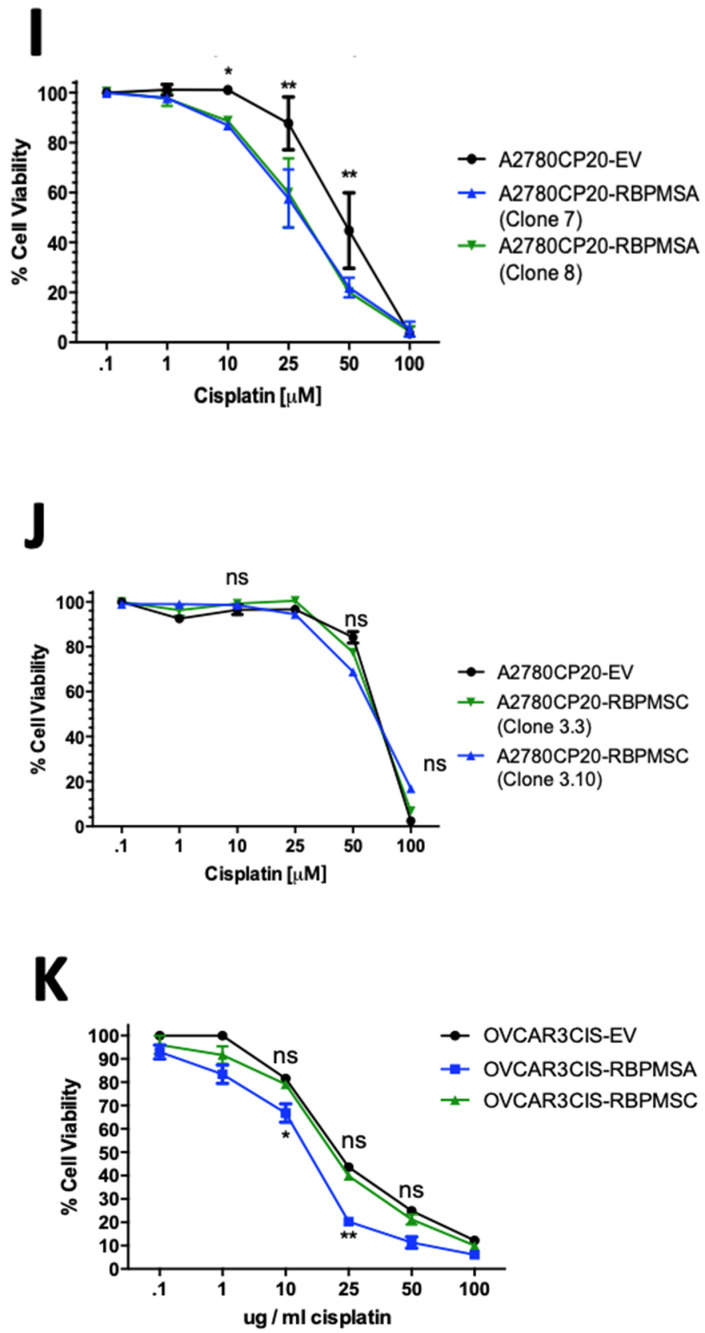

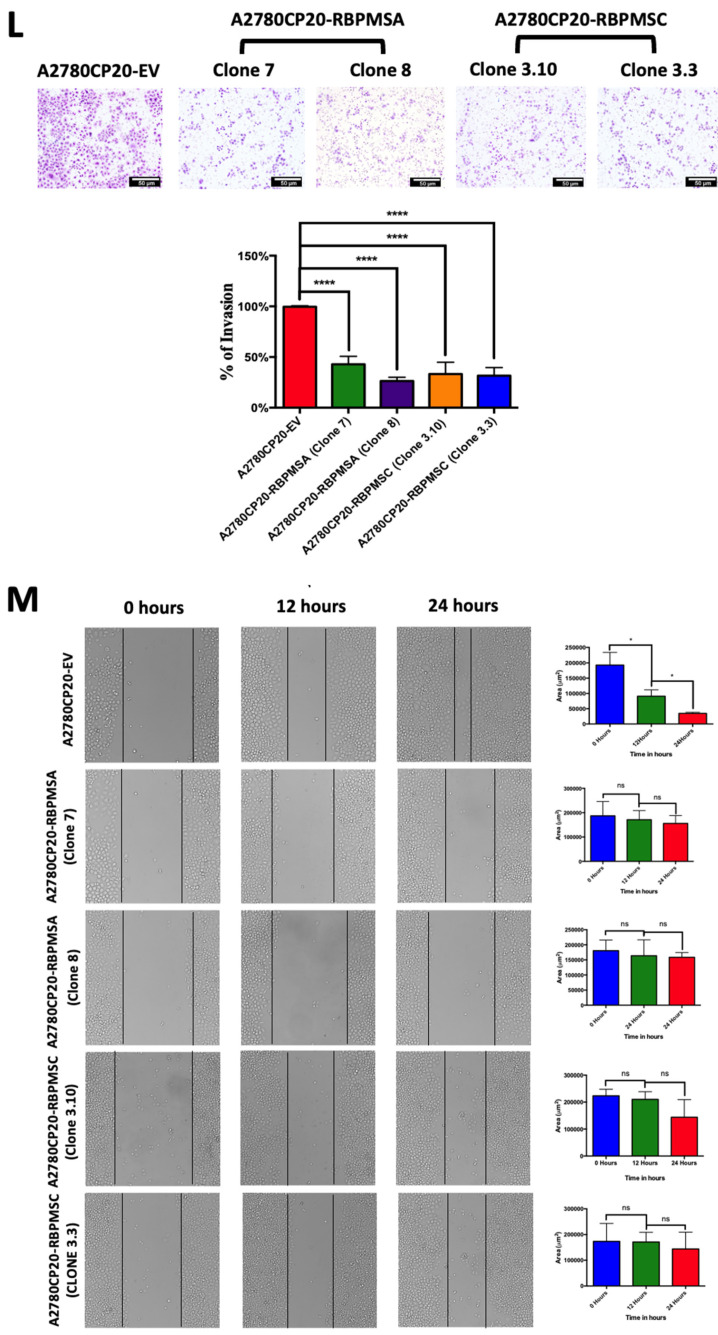
Effect of RBPMSA and RBPMSC overexpression in cell growth, proliferation, invasion, and migration. (**A**,**B**) Colony formation assay. Percentages of clonogenicity were calculated relative to A2780CP20-EV cells. (**C**,**D**) Colony Size. Percentages of size measures were calculated relative to A2780CP20-EV cells. (**E**) OVCAR3CIS were transiently transfected with RBPMSA, RBPMSC, or an empty vector. A concentration of 50 μg of protein extracts was used to perform Western blots and β-actin was used as a loading control. The increases in molecular weight of the RBPMSA and RBPMSC bands correspond to the 12 extra aminoacids of the DDK-Tag sequence. (**F**) Colony formation assay. Percentages of clonogenicity were calculated relative to OVCAR3CIS-EV cells. (**G**) Colony Size. Percentages of size measures were calculated relative to OVCAR3CIS-EV cells. (**H**) Cell growth curves cells (2.0 × 10^4^ cells/mL) were seed in a 10 cm Petri and detached with Trypsin (0.25%) at 37 °C, staining with 0.5% trypan blue solution, and counted in triplicates every 24 h for 96 h after plating using a hemocytometer. Viability Assays. (**I**,**J**) A2780CP20-EV, A2780CP20-RBPMSA (clones 7 and 8), and A2780CP20-RBPMSC (clones 3.10 and clones 3.3) and (**K**) OVCAR3CIS-EV, OVCAR3CIS-RBPMSA, and OVCAR3CIS-RBPMSC transiently transfected cells (all at 3 × 10^4^ cell/mL) were exposed to different concentrations (0.1 μg/mL, 1.0 μg/mL, 10 μg/mL, 25 μg/mL, 50 μg/mL and 100 μg/mL) of cisplatin for 72 h. Percentages of cell viability were calculated relative to EV cells. (**L**) Cell invasion. Percentages of invasion were calculated relative to A2780CP20-EV cells. Bars represent the means of triplicates ± S.E.M. (**M**) Representative images of scratch wound healing assay at 0, 12, and 24 h. Bars in the graph of (**L**) represent the area in μm^2^ of the middle area of the cell migration images. Bars: mean of triplicates ± S.E.M. * *p* < 0.05, ** *p* < 0.01, *** *p* < 0.001, **** *p* < 0.0001 and ns = not significant.

Seeing that RBPMSA and RBPMSC were dramatically reduced in cisplatin resistant compared with cisplatin sensitive cells, we wanted to study the biological consequences of overexpressing each RBPMSA and RBPMSC in A2780CP20 and OVCAR3CIS cells. A2780CP20 cells were stable transfected and OVCAR3CIS were transiently transfected with RBPMSA or RBPMSC plasmids. Figure 1H,I is a Western blot showing the protein levels of A2780CP20-RBPMSA (21.8 kDa) or A2780CP20-RBPMSC (24.2 kDa) clones. Figure 1J,K are densitometric analysis of the band intensities of the Western blot images. Original images of the Western blots were included in Supplementary Figure S2. In clonogenic assays, we observed a significant reduction in the number of colonies formed by cells that overexpressed RBPMSA or RBPMSC (*** *p* < 0.001 and **** *p* < 0.0001, respectively) compared with A2780CP20-EV clones (Figure 2A,B). Moreover, the size of the colonies (Figure 2C,D) formed by A2780CP20-RBPMSA or A2780CP20-RBPMSC overexpressing clones were significantly smaller when compared with A2780CP20-EV clones (**** *p* < 0.0001 and ** *p* < 0.01, respectively). Figure 2E is a Western blot showing the overexpression of each RBPMS isoform in OVCAR3CIS. Original western blot image in Supplementary Figure S3a. The bands close to 31 kDa correspond to RBPMSA and the band close to 34 kDa corresponds to OVCAR3CIS-RBPMSC. These increases in molecular weight are due to the extra 12 aminoacids of a DDK-Tag sequence included in the pCMV6 vector. Overexpression of RBPMSA and RBPMSC in OVCAR3CIS resulted in a significant reduction in the number of colonies and the colony sizes compared with OVCAR3CIS-EV clones (*** *p* < 0.001 **** *p* < 0.0001) (Figure 2F,G). We tested the effect of RBPMSA and RBPMSC overexpression on cell growth rates. Figure 2H shows that both A2780CP20-RBPMSA and A2780CP20-RBPMSC grew slower as compared with the A2780CP20-EV clones. Together, these results suggest that increased levels of RBPMSA and RBPMSC reduce cell proliferation in cisplatin resistant ovarian cancer cells.

### 2.3. RBPMSA Overexpression Increased the Sensitivity of Ovarian Cancer Cells to Cisplatin Treatment

We next aimed to determine whether overexpression of RBPMSA or RBPMSC splice variants increased the sensitivity of ovarian cancer cells to cisplatin treatment. A2780CP20-RBPMSA (clone 7 IC50: 29.77 µg/mL and clone 8 IC50: 30.03 µg/mL) showed an increase in cisplatin sensitivity compared with the control A2780CP20-EV (IC50: 57.73 µg/mL) (Figure 2I). However, A2780CP20-RBPMSC (clone 3.3 IC50: 53.42 µg/mL and clone 3.10 IC50: 56.69 µg/mL) did not show a significant increase in cisplatin sensitivity compared to A2780CP20-EV (IC50: 58.99 µg/mL). (Figure 2J). Similar tendency was observed in OVCAR3CIS cells as obtained overexpression of RBPMSA in these cells exhibited an increase in cisplatin sensitivity (IC50: 18.89 µg/mL) compared with OVCAR3CIS-EV (IC50: 33.01 µg/mL) cells. OVCAR3CIS cells overexpressed with RBPMSC overexpression did not show increases in cisplatin sensitivity (IC50: 31.69 µg/mL) compared to OVCAR3CIS-EV cells (Figure 2K). Together, these results suggested that RBPMSA but not RBPMSC levels increase the sensitivity of ovarian cancer cells to cisplatin treatment.

### 2.4. RBPMSA and RBPMSC Overexpression Decreased the Migration and the Invasion Ability of Ovarian Cancer Cells

RBPMS knockout has been associated with increased invasion ability in ovarian cancer [17]. We assessed the effect of RBPMSA and RBPMSC overexpression in the migration and invasiveness potential in ovarian cancer cells. In a transwell invasion assay, we confirmed that A2780CP20-RBPMSA decreased the invasion capacity of the cells in clones 7 (**** *p* <0.0001) and 8 (**** *p* <0.0001) when compared with the A2780CP20-EV clone. Similarly, results were observed in A2780CP20-RBPMSC clones 3.3 (**** *p* <0.0001) and 3.10 (**** *p* <0.0001). Remarkably, the number of invaded cells in with each A2780CP20-RBPMSA and A2780CP20-RBPMSC clones was 50% less than with the A2780CP20-EV clones (Figure 2L). In the wound healing assays, we observed that the A2780CP20-RBPMSA and A2780CP20-RBPMSC clones lost the ability to migrate, as shown in Figure 2M. Significant migration of cells was noted only with the A2780CP20-EV clones (Figure 2M). This data suggests that RBPMSA and RBPMSC significantly reduced the invasive and migration ability of cells when compared to A2780CP20-EV clones.

### 2.5. RBPMSA and RBPMSC Overexpression Decreased the Senescence-Associated β-Galactosidase Levels

**Figure 3:**
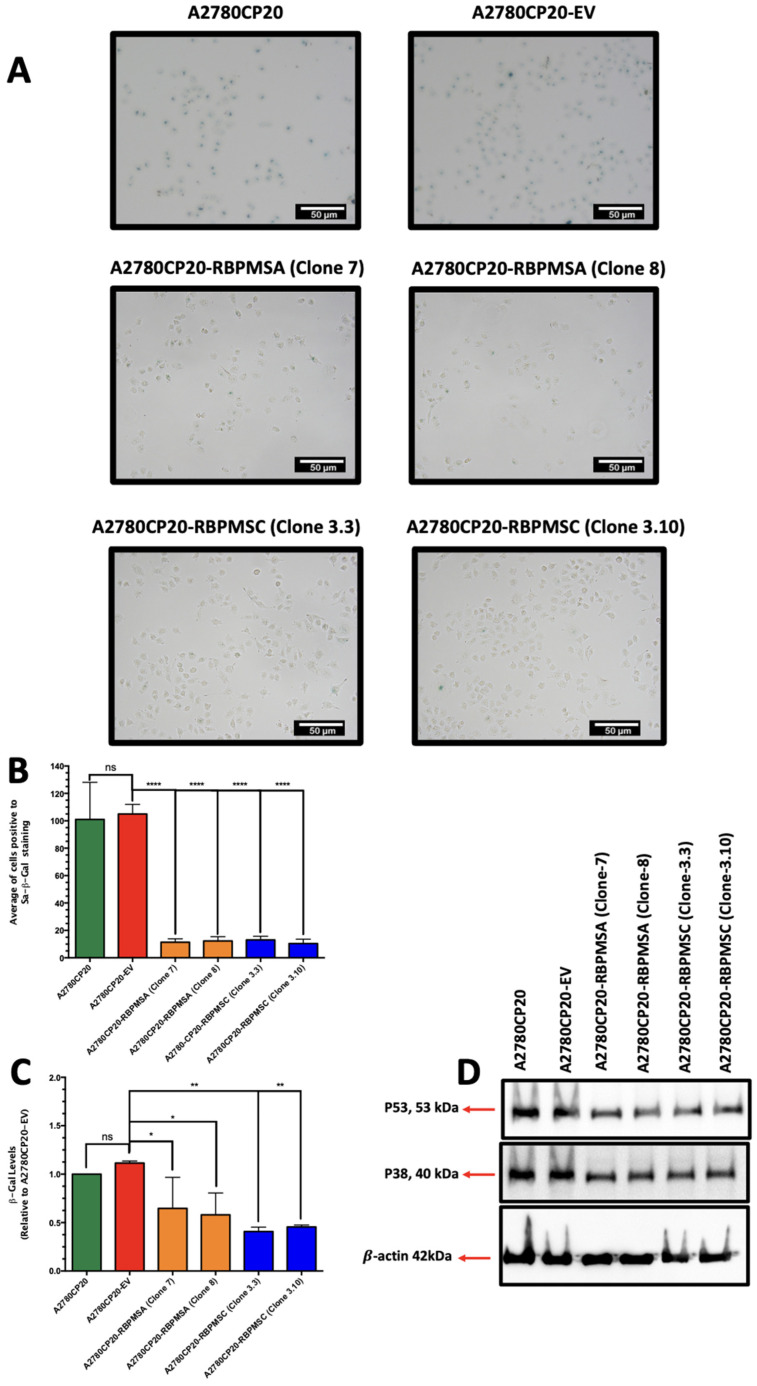

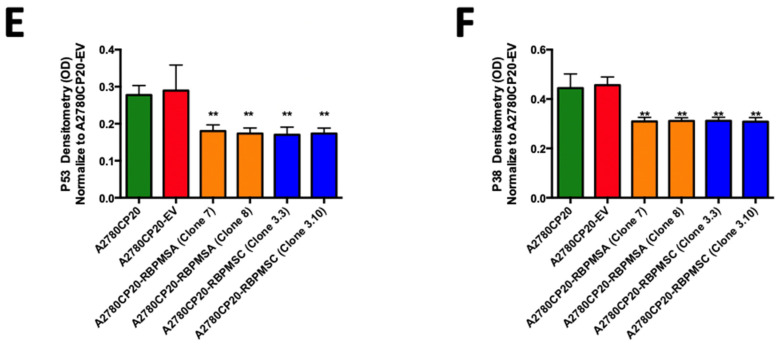
Effect of RBPMSA and RBPMSC Overexpression on Senescence. (**A**) Representative images of SA-β-Gal-stained cells. (**B**) Quantification of the positive SA-β-Gal-stained cells. Images scale bar: 50 µm (bars: five microscopic fields per condition). Staining were done acording to manufacturer specification. * *p* < 0.05, ** *p* < 0.01, **** *p* < 0.0001 and ns = no significant. (**C**) Cells (1 × 10^4^ cells/mL) were plated in Petri dishes. Next day, cells were rinsed with PBS, and protein extracts were prepared at 1 µg/mL protein concentration. Senescence-associated β-galactosidase activity (SA-β-gal) was assessed via fluorescence. β-galactosidase levels were calculated relative to A2780CP20-EV cells. Averages ± SEM are shown for three independent experiments. (**D**) Western blots were performed with 50 µg of protein extracts and β-actin was used as a loading control. (**E**,**F**) Densitometry analysis of band intensities shown in (**D**). Fold changes in protein levels were calculated relative to the A2780CP20-EV clones (** *p* < 0.01).

Evidence indicates that the acquisition of drug resistance by cancer cells is accompanied by senescence phenotypes [20]. Thus, we investigated if either RBPMSA or RBPMSC overexpression promote senescence phenotypes in ovarian cancer cells. Lower SA-β-Gal positive staining cells were observed in A2780CP20-RBPMSA or A2780CP20-RBPMSC clones compared with A2780CP20-EV clones (Figure 3A). Figure 3B shows the number of SA-β-Gal-positive cells registered in Figure 3A, which confirmed our observations. We also quantify the senescence-associated beta-galactosidase (β-Gal) levels in A2780CP20-EV, A2780CP20-RBPMSA, and A2780CP20-RBPMSC clones. Smaller β-Gal levels were detected in A2780CP20-RBPMSA (* *p* < 0.1) or A2780CP20-RBPMSC (** *p* < 0.01) clones compared with A2780CP20-EV clones (Figure 2C). Increased levels of p21, p38, and p53 are associated with senescence phenotypes of cancer cells [20]. Figure 3D shows that the p53 and p38 protein levels were reduced in A2780CP20-RBPMSA and A2780CP20-RBPMSC clones as compared with A2780CP20-EV clones or with A2780CP20 cells. P21 protein expression was not observed in A2780CP20 cells or the clones. Original images of the Western blots were included in Supplementary Figure S3b.

### 2.6. Effects of Subcutaneous Implantation of RBPMSA and RBPMSC Overexpressing Cells in an Ovarian Cancer Mouse Model

**Figure 4:**
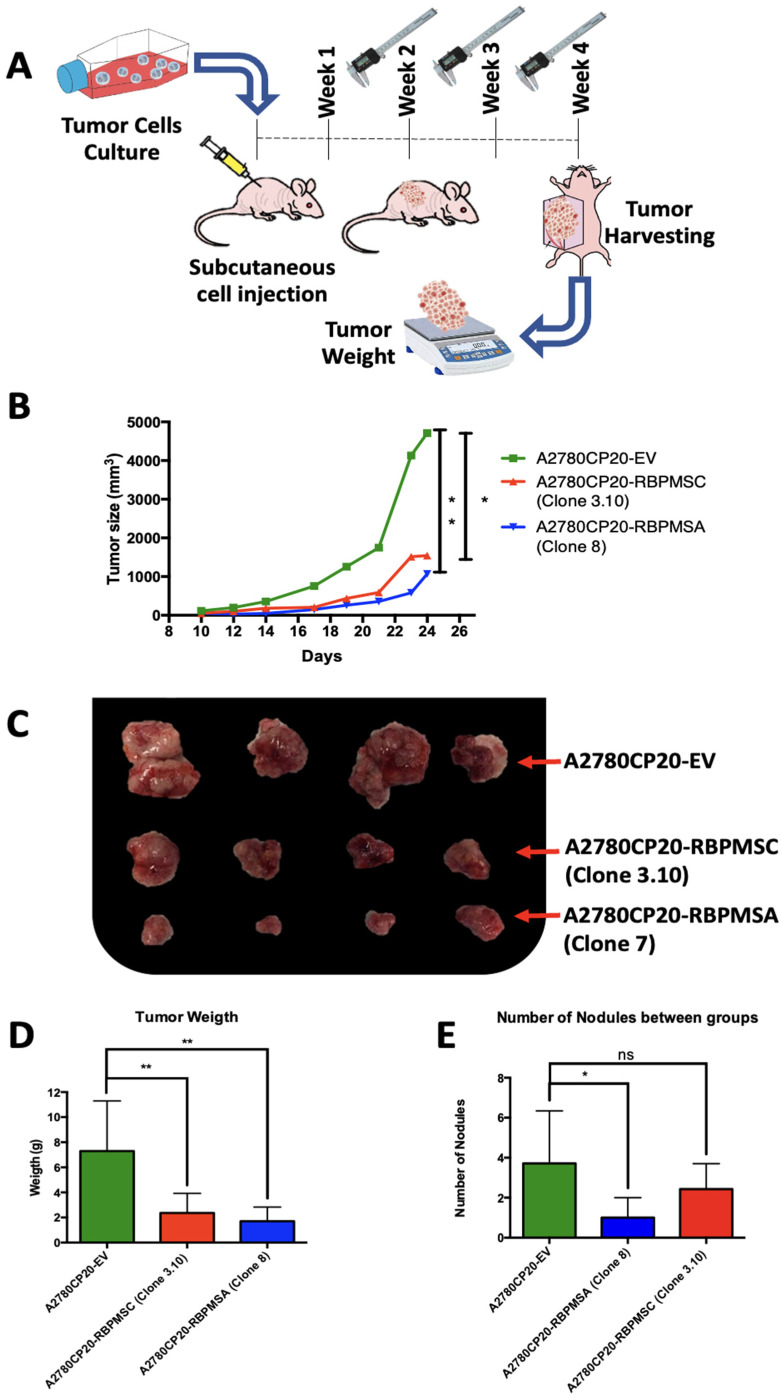

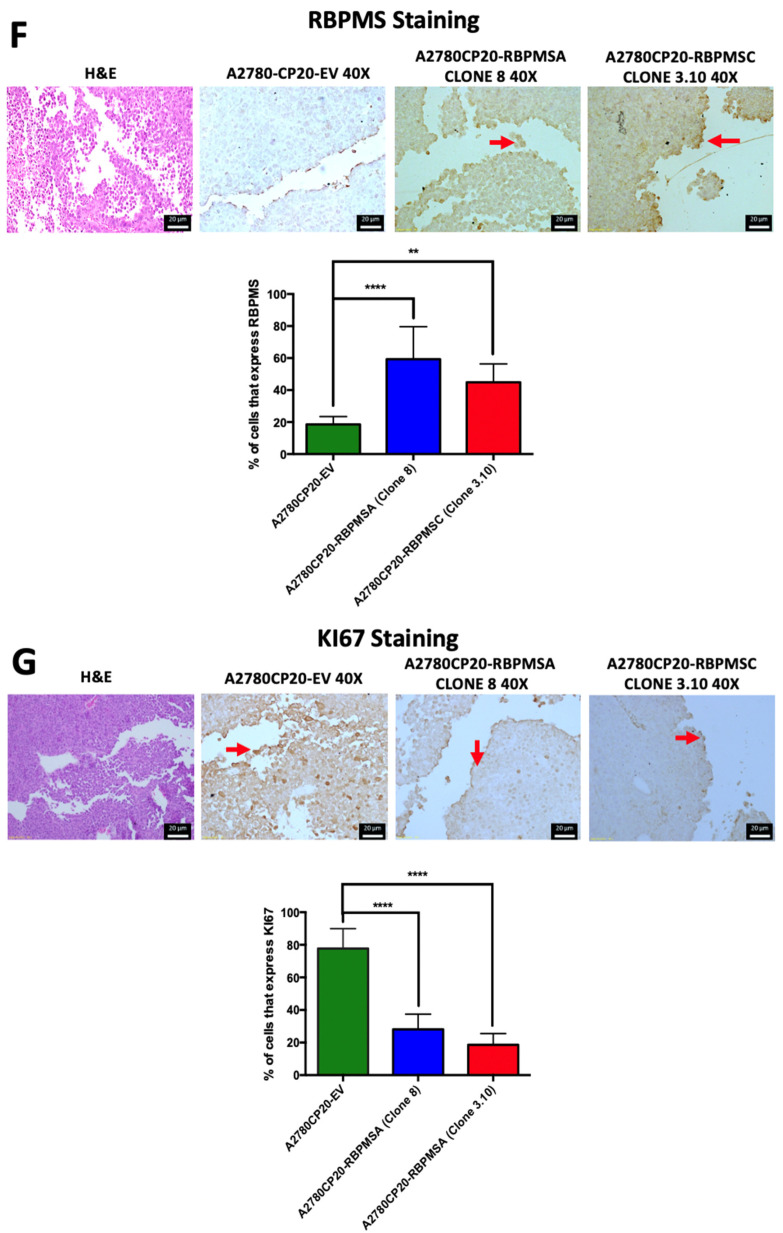

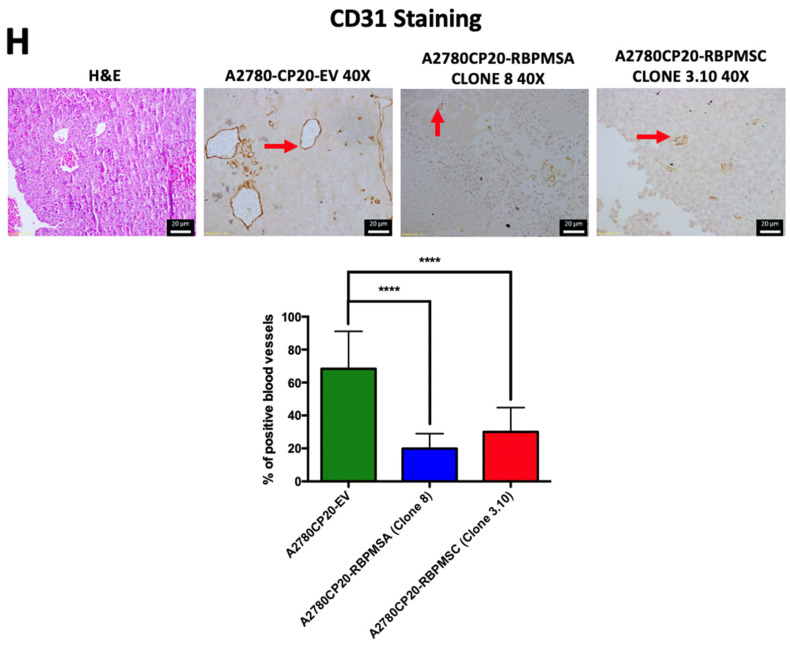
Effect of RBPMSA and RBPMSC overexpression on in vivo tumor growth. (**A**) Graphical image of the in vivo experiment. Mice were subcutaneously injected with A2780CP20-RBPMSA, A2780CP20-RBPMSC, and A2780CP20-EV (Number of mice, N = 7 per group). The tumor growth was monitored with a Vernier caliper three times per week. (**B**) Tumor size measurements. (**C**) A visual image of tumor size at the end of the experiment. (**D**) Tumor weight (**E**) Number of nudes. (**F**–**H**) Representative images of IHC experiments for RBPMS protein levels, proliferation (KI-67), and blood vessel formation (CD31). Microscopy images were taken at 20× (Supplementary Figure S4) and 40× magnification. Red arrow shows a positive cell staining signal with respective antibody. Quantification of RBPMS, CD31, and KI67 staining was determined by Image J software. Data is presented as the mean ± SEM of staining relative to A2780CP20-EV. Significant variations between groups and A2780CP20-EV control were determined by Student’s *t*-test. * *p* < 0.05, ** *p* < 0.01, **** *p* < 0.0001 and ns = no significant.

We then assessed the effect of RBPMSA or RBPMSC on tumor progression in a subcutaneous ovarian cancer mouse model. A2780CP20-RBPMSA (clone 8), A2780CP20-RBPMSC (clone 3.10), and A2780CP20-EV cells (see Figure 1H,I) were subcutaneously injected into the right dorsal flank of female athymic mice (Figure 4A). Seven days after cell implantation, tumor size was measured with a Vernier caliper three times per week for three weeks. Figure 4B shows that the tumors of A2780CP20-RBPMSA and A2780CP20-RBPMSC clones grew slower as compared with tumors of A2780CP20-EV clones. At the end of the experiment, the difference in the tumor sizes between A2780CP20-RBPMSA or A2780CP20-RBPMSC and the controls group (A2780CP20-EV) were statistically significant (** *p* < 0.01, and * *p* < 0.05, respectively). Figure 4C shows the appearance of the tumors at the end of the experiment. Additionally, tumor weight and the number of nodules showed a statistically significant difference between the A2780CP20-RBPMSA or A2780CP20-RBPMSC groups and the A2780CP20-EV group (Figure 4D,E).

Then, we performed IHC studies to measure the RBPMS protein levels, the tumor cell proliferation rates (Ki67), and the blood vessel formation (CD31) in tissue sections of the mice tumors (Figure 4F–H). As expected, the RBPMS immunoreactivity was significantly higher for A2780CP20-RBPMSA (*** *p* < 0.0001) or A2780CP20-RBPMSC (** *p* < 0.01) tumor tissues compared with A2780CP20-EV tumor tissues (Figure 4F). We found that tumor tissues of A2780CP20-RBPMSA or A2780CP20-RBPMSC overexpressing cells had significantly lower percentage of Ki67 positive stained cells (proliferative index) compared with tumor tissues of 2780CP20-EV cells (Figure 4G). Tumor tissue sections were also stained with the endothelial CD31 marker to assess angiogenesis. As shown in Figure 4H, tissues of A2780CP20-RBPMSA (**** *p* < 0.0001) or A2780CP20-RBPMSC (**** *p* < 0.0001) had a significantly lower percentage of positive blood vessels as compared with A2780CP20-EV tumor tissues.

### 2.7. Identification of RBPMSA and RBPMSC Downstream Signaling Pathways by RNAseq

**Figure 5:**
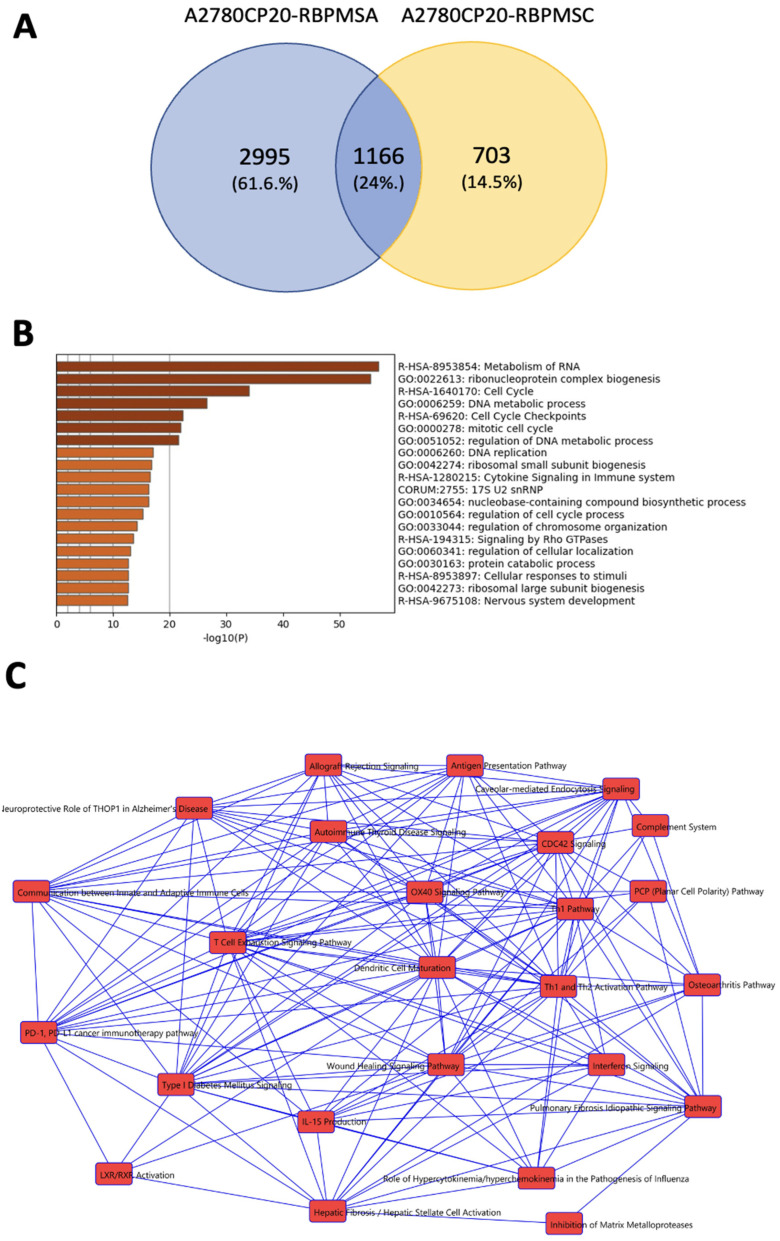

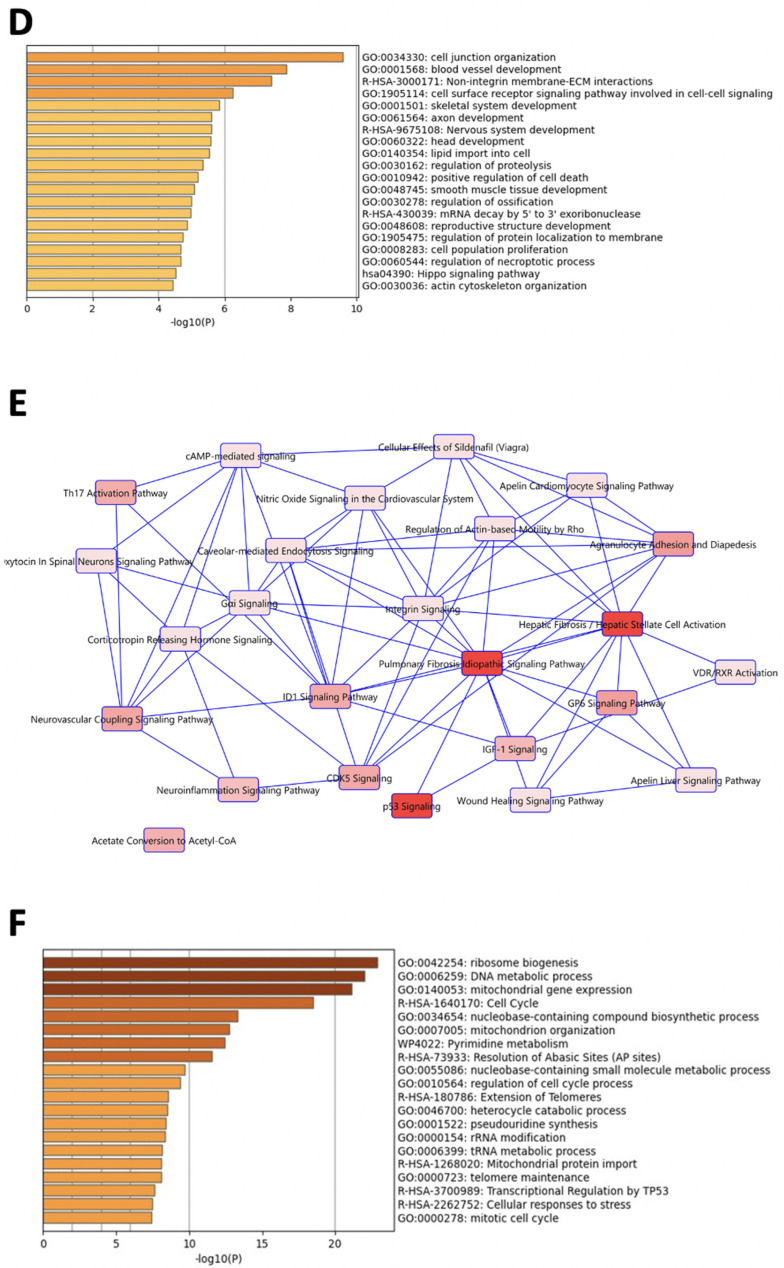

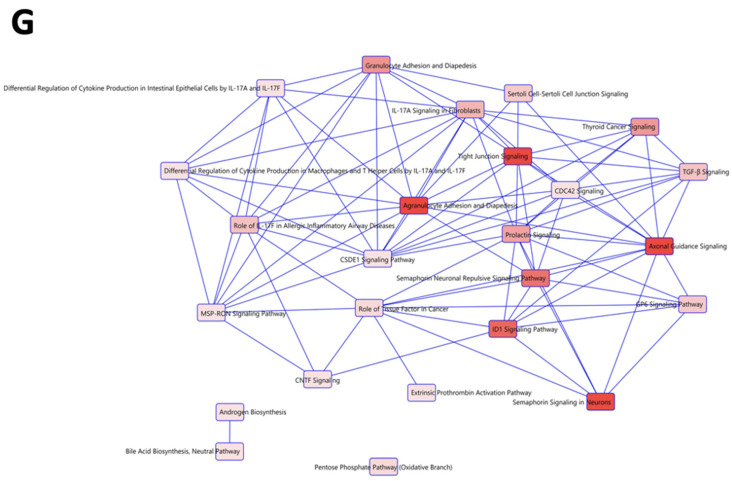
Ingenuity pathway analysis (IPA) and functional enrichment analysis of top deregulated transcripts in RBPMSA and RBPMSC clones. (**A**) Venn diagram showing that 2995 RNA transcripts were deferentially abundant in RBPMSA clones, 703 in RBPMSC clones, and 1166 were common to both, A2780CP20-RBPMSA and A2780CP20-RBPMSC clones. (**B**) The 20 top most significant (*p*-value ≤ 0.05) enriched ontology clusters by Gene ontology analysis of functional enrichment in A2780CP20-RBPMSA clones. (**C**) Interaction network of the top canonical pathways identified in the A2780CP20-RBPMSA clones. (**D**) The 13 top most significant (*p*-value ≤ 0.05) enriched ontology clusters by Gene ontology analysis of functional enrichment in A2780CP20-RBPMSC clones. (**E**) Interaction network of the top canonical pathways identified in the A2780CP20-RBPMSC clones. (**F**) The 20 top most significant (*p*-value ≤ 0.05) enriched ontology clusters by Gene ontology analysis of functional enrichment in common transcripts between A2780CP20-RBPMSA and A2780CP20-RBPMSC clones. (**G**) Interaction network of the top canonical pathways identified in the common transcripts between A2780CP20-RBPMSA and A2780CP20-RBPMSC clones.

To further identify the downstream effectors of each RBPMS splice variant, we performed RNAseq in total RNA extracted from A2780CP20-EV, A2780CP20-RBPMSA, and A2780CP20-RBPMSC overexpressed clones. We initially identified 16,968 RNA transcripts in the A2780CP20-RBPMSA sample and 16,717 in the A2780CP20-RBPMSC sample (See Supplementary Table S1). Further filtering using a cut-off *p*-value < 0.05 and fold change ≥ |2.0| reduced the list of significantly expressed RNA transcripts to 4161 in A2780CP20-RBPMSA and 1869 for A2780CP20-RBPMSC samples (Supplementary Table S2). A Venn diagram showed that 2995 RNA transcripts were exclusive to A2780CP20-RBPMSA and 703 for A2780CP20-RBPMSC. Moreover, 1161 transcripts were shared (common) by the two RBPMS splice variants (Figure 5A). Table 1 includes the top 20 (10 upregulated and 10 downregulated, selected by fold change) differentially regulated transcripts in the A2780CP20-RBPMSA overexpression clones, and Table 2 shows the top 13 differentially regulated transcripts in the A2780CP20-RBPMSC overexpression clones (only three genes were significantly downregulated in the A2780CP20-RBPMSC clones). The RNAseq results were validated using real time PCR. The validation included the top 10 differentially expressed transcripts (seven upregulated and three downregulated) in RBPMSA vs. A2780CP20-EV. As it is shown in Supplementary Table S3 and Figure S5, nine (of the 10 genes validated by PCR) correlated well with the RNAseq results. The same validation was performed for the top eight differentially expressed transcripts in RBPMSC vs. A2780CP20-EV (five upregulated and three downregulated). The PCR data showed that five out of eight genes correlated well with the RNAseq data (Supplementary Table S4 and Figure S6). Deregulation of most of these genes has already been associated with cancer progression, metastasis, and immune system response [10,17,21,22,23]. For example, interferon induced protein 44 (IFI44), one of the most increased transcripts upon A2780CP20-RBPMSA overexpression, has been linked to the suppression of the proliferation of human melanoma cell lines [24] as well as immune response to autoimmune disease [25]. Interestingly, two long non-coding RNAs (lncRNAs), LINC01504 (increased) and SNORD99 (decreased), were regulated in A2780CP20-RBPMSA clones. For A2780CP20-RBPMSC, Calbindin 2 (CALB2), the second most increased transcript, has been linked as an important mediator of 5-FU-induced cell death [26]. Moreover, in the list of common transcripts shared by A2780CP20-RBPMSA and A2780CP20-RBPMSC clones, we identified ANKRD33B, which increase in CpG methylation has associated with oral and pharyngeal squamous cell carcinoma cell lines and primary non-neoplastic oral epithelial cells [27] and RAD51 which recently received considerable attention due to its function in tumor progression and its decisive role in tumor resistance to chemotherapy. Moreover, RAD51 plays a role in maintaining the stability of a cell’s genetic information mediating the DNA damage repair [28] (Table 3).

To better examine the interaction networks of RBPMS downstream genes, the lists with the 2995 transcripts of A2780CP20-RBPMSA, the 703 of A2780CP20-RBPMSC, and the common 1161 transcripts were subjected to functional enrichment using Metascape via Gene Ontology (GO) and the Kyoto Encyclopedia of Genes and Genomes (KEGG) and uploaded into the Ingenuity Pathway Analysis (IPA) software [74]. Among the top 20 most significantly (*p*-value ≤ 0.05) enriched ontology clusters of A2780CP20-RBPMSA, the most relevant clusters included the metabolism of RNA, ribonucleoprotein complex biogenesis, and cell cycle (Figure 5B). Figure 5C includes the interactions between the top canonical pathways identified in the A2780CP20-RBPMSA clones. The top canonical pathways were the hepatic fibrosis/hepatic stellate cell activation, inhibition of matrix metalloproteases, wound healing signaling, CDC42 signaling, and PD-1-PD-L1 cancer immunotherapy pathway. The top five networks in terms of the number of genes per pathway are depicted in Supplementary Table S5. These pathways included Cancer, Cardiovascular System Development and Function Organismal Development (31 genes), Cell Cycle, Cellular Development, Cellular Growth and Proliferation (25 genes), Antimicrobial Response, Inflammatory Response, and Organismal Injury and Abnormality (55 genes) pathways.

Similarly, for the A2780CP20-RBPMSC, the top 20 most significantly (*p*-value ≤ 0.05) enriched ontology clusters included the cell junction organization, blood vessel development and non-integrin membrane-ECM interactions (Figure 5D). Figure 5E includes the interaction network of the top canonical pathways identified for A2780CP20-RBPMSC clones. The top canonical pathways were the P53 signaling, hepatic fibrosis/hepatic stellate cell activation, pulmonary fibrosis idiopathic signaling pathway, CDK5 signaling pathway and IGF1 signaling pathway. The networks in terms of the number of genes per pathway for A2780CP20-RBPMSC are depicted in Supplementary Table S3. These pathways included cardiovascular system development and function, cell to cell signaling and interaction, cellular movement (2 genes), organ morphology, reproductive system development and function, tissue development (3 genes), antimicrobial response, cell cycle, and survival (2 genes) pathways.

We also performed similar analysis with the common transcripts regulated in both A2780CP20-RBPMSA and A2780CP20-RBPMSC clones. Among the top 20 most significantly (*p*-value ≤ 0.01) enriched ontology clusters, the most relevant included ribosome biogenesis, DNA metabolic process, and mitochondrial gene expression (Figure 5F). Figure 5G includes the interaction between the top canonical pathways identified with the common transcripts between A2780CP20-RBPMSA and A2780CP20-RBPMSC clones. The top canonical pathways involved TGF-β signaling, role of tissue factor in cancer, and cytokine production in macrophages and T helped cells by IL-17A and IL-17F. The networks shared by A2780CP20-RBPMSA and A2780CP20-RBPMSC in terms of the number of genes per pathway included: Cancer, Cardiovascular Disease Hematological System Development and Function (2 genes), Cell to Cell Signaling and Interaction, Cellular Development, Cellular growth, and Proliferation (2 genes), Cancer, Cellular Movement, Organismal Injury and Abnormality (2 genes) (Supplementary Table S3).

### 2.8. Prognostic Value of RBPMSA and RBPMSC Downstream Effectors

**Figure 6:**
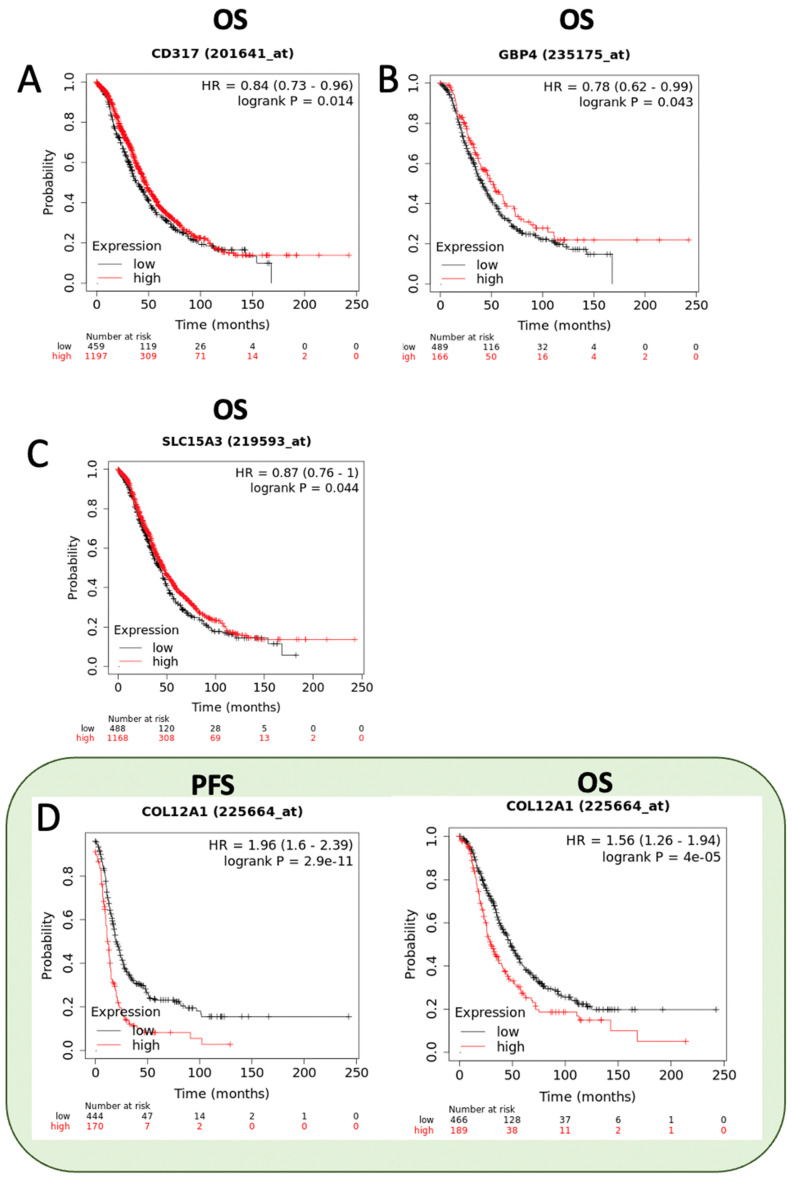

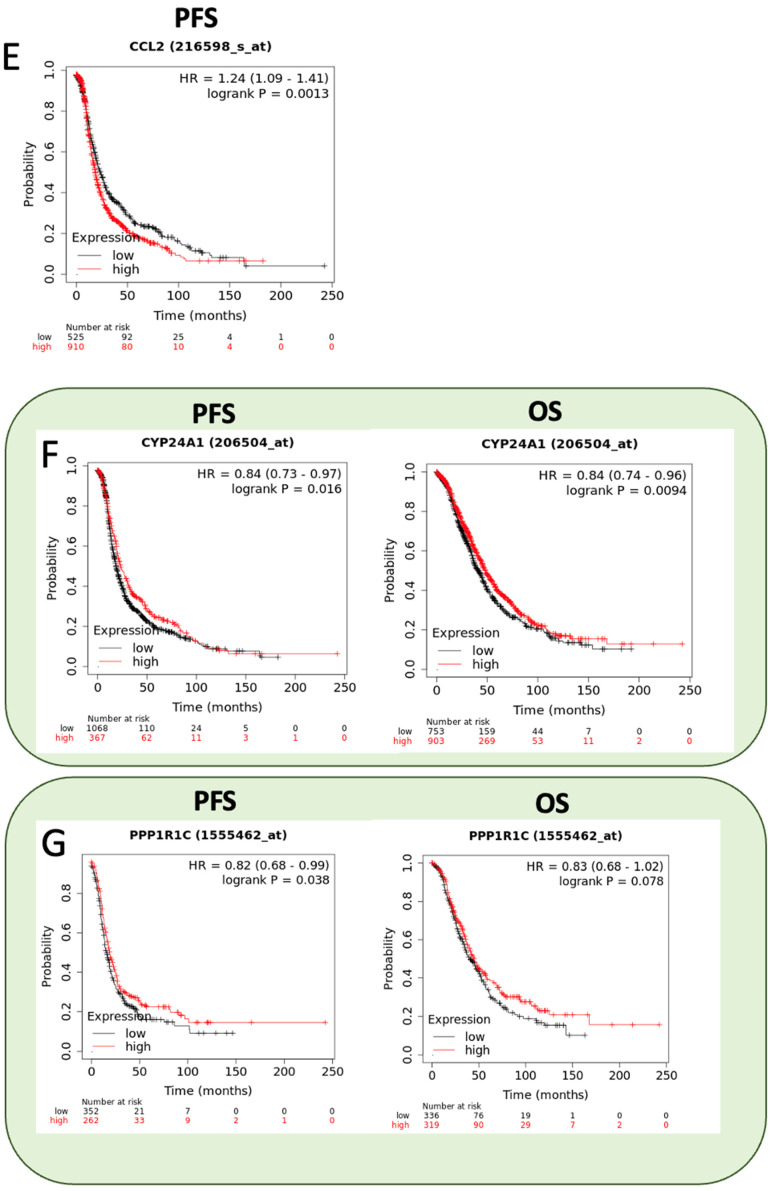

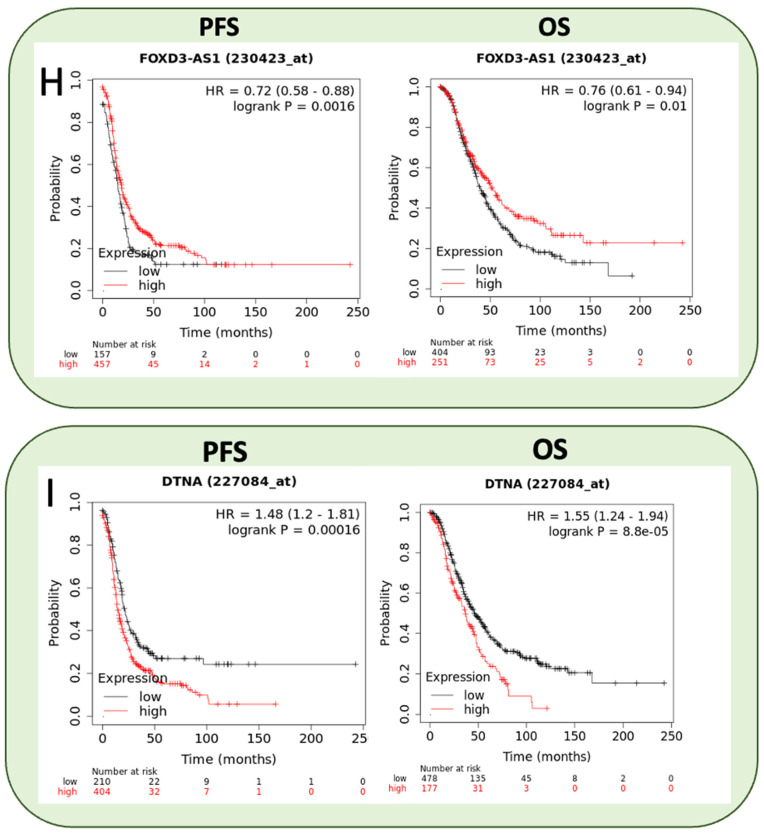
KM survival curves. KM plots for ovarian cancer patients were generated using the KM plotter database. The OS and PFS of the ovarian cancer patients were stratified based on the median RNA expression levels for each gene (**A**) CD3117 (**B**) GBP4 (**C**) SLC15A3 (**D**) COL12A1 (**E**) CCL2 (**F**) CYP24A1 (**G**) PPP1R1C (**H**) FOXD3-AS1 (**I**) DTNA.

To assess the clinical relevance of the top differentiated abundant transcripts (see Table 1, Table 2 and Table 3) identified by RNAseq in A2780CP20-RBPMSA and A2780CP20-RBPMSC clones, we interrogated the Kaplan-Meier plotter data base (KM plotter). Ovarian Cancer KM plotter includes data from “The Cancer Genome Atlas” (TCGA), Gene Expression Omnibus (GEO), and European Genome Archive (EGA) for a total of 1436 ovarian cancer samples [75]. Overexpression of RBPMSA in A2780CP20 cell line increased the RNA levels of BST2 (also known as CD317), GBP4, and SLC15A. In agreement with these results, higher RNA expression levels of these genes were associated with better prognosis of the disease (OS; HR < 1) (Figure 6A–C). On the other hand, overexpression of RBPMSA reduced the expression levels of COL12A1 and CCL2. Again, KM plotter data analysis showed that lower expression levels of COL12A1 were associated with longer PFS (HR > 1) and better prognosis (OS; HR > 1) (Figure 6D,E). High expression levels of CYP24A1, PPPIRIC, and FOXD3-AS1, detected in A2780CP20-RBPMSC clones, were associated with longer PFS (HR < 1) and better prognosis (OS; HR < 1) of ovarian cancer patients (Figure 6F–H). Moreover, decreased levels of DTNA in A2780CP20-RBPMSC clones were associated with longer PFS (HR > 1) and better prognosis (OS; HR > 1) in patients (Figure 6I).

## 3. Discussion

Accumulating evidence indicate that RBPMS is a key RNA binding protein involved in the metabolism of RNA molecules. Several RBPMS splice variants are originated from a single primary transcript; three of them have been reported at the protein level: RBPMSA, RBPMSB, and RBPMC. It is speculated that each RBPMS splice variant binds and process its own group of RNAs [76]. We previously reported that CRISPR-mediated RBPMS knockdown reduced the sensitivity of ovarian cancer cells to cisplatin treatment [17]. However, the role of each RBPMS splice variant in ovarian cancer cells had not been studied previously. Here, we reported for the first time that the mRNA and protein levels of RBPMSA and RBPMSC are reduced in cisplatin-resistant ovarian cancer cell lines compared to their cisplatin-sensitive counterparts. Not detectable mRNA and protein levels of RBPMSB were observed in cisplatin sensitive and cisplatin resistant ovarian cancer cells. Overexpression of RBPMSA and RBPMSC into cisplatin-resistant ovarian cancer cell line A2780CP20 decreased cell growth, migration, invasion, and reduced senescence associated with β-Galactosidase levels. Moreover, RBPMSA, but not RBPMSC, increased the sensitivity of ovarian cancer cells to cisplatin treatment. Similar results were obtained by using the HGSOC cell line OVCAR3CIS.

Nakagaki et al. showed that RBPMS is a master splicing regulator in vascular smooth muscle cells (SMCs) [76]. Knockdown of RBPMS in differentiated smooth muscle cell line PAC1 led to changes in mRNA abundance levels, promoting a differentiated alternative splicing program [76]. Also, Rastgoo et al. reported that RBPMS restauration by overexpressing miR-138 re-sensitized multiple myeloma cells to the proteasome inhibitor bortezomib (BTZ) [77]. These two reports interrogated only the canonical RBPMS (RBPMSA, also known as RBPMS1). Fu et al. showed that decreased expression of RBMSA and RBPMSC promoted cell growth, survival and drug resistance of breast cancer cells [18]. The exact molecular mechanism by which each RBPMS splice variant exerts its biological effects are currently unknown; but Fu et al. reported that RBPMSA and RBPMSC bind and repress AP-1 transcription factor [18]. Also, Sun et al. reported that overexpression of RBPMS enhanced Smads’ transcriptional activity in human embryonic kidney 293T cells. Sun et al. showed that interaction of RBPMS with TGF-β receptor type I increased phosphorylation of Smad2 and Smad3, and promoted the nuclear accumulation of the Smads proteins [19]. Therefore, each RBPMS splice variant could bind to key transcription factors and/or modify its own groups of RNA transcripts. These hypotheses require further investigation.

We observed that overexpression of RBPMSA and RBPMSC in A2780CP20 cells decreased the senescence-associated β-Gal levels of these cells. This effect was accompanied by the increased protein levels of p53 and p38. Curiously, A2780CP20 cells do not express p21 [20]. Decreased expression of p21 and p53, two key cell cycle progression regulators had also been associated with a senescence phenotype of cancer cells [78]. Santana et al. studied the effect of Enolase-1 (ENO1) in ovarian cancer cells and observed that decreased expression of ENO1 promoted glucose accumulation, induced senescence, increased the p53 protein levels, and promoted cisplatin resistance of ovarian cancer cells [20]. In addition, the mitogen activated protein kinase p38 activates a wide range of substrates that include transcription factors, protein kinases, and nuclear proteins, leading to diverse responses, including senescence and chemoresistance processes [79]. Guo et al. studied the effect of phosphorylated p38 in the human gastric cancer cells SGC7901/VCR cell line and observed that inhibition of p38 with the small molecule inhibitor SB203580 reversed the multidrug resistance of these cells [79]. Although evidence indicates that chemotherapy induces a beneficial short term senescence stage during chemotherapy treatment, it could promote changes in gene expression leading to reprogramming in cancer cells. Reprograming of these cancer cell populations in a tumor could be an adaptive pathway that later generates more aggressive and highly drug-resistant phenotype clones, a characteristic of the tumor heterogeneity [80]. Senescent cells are characterized by altered cell metabolism, increased lysosomal capacity, and they have the potential to secrete different molecules (i.e., pro-inflammatory cytokines and growth factors) to the microenvironment (TME) [78,81]. The production of all these molecules is known as the senescence associated secretory phenotype (SASP). The SASP promote cell proliferation, induce epithelial to mesenchymal transition EMT [82], enhance invasion [83], and promotes chemoresistant and radioresitant phenotypes [84]. Thus, increasing the RBPMS levels could have the potential to take out cells of senescence stages, and reduce the cell growth and proliferation of cisplatin resistant ovarian cancer cells. These hypothesis should be further investigated.

Reduced protein levels of RBPMS have been documented in bladder cancer [85], multiple myeloma [77], ovarian cancer [17], and osteoarthritic cartilage cell lines [86]. However, in these studies, only RBPMSA (RBPMS1) was studied. By using a subcutaneous ovarian cancer mouse model, we observed that increased expression of RBPMSA and RBPMSC resulted in smaller tumors compared with controls. This effect was more noticeable with tumors overexpressing the RBPMSA isoform. Tumors overexpressing the RBPMSA isoform also had reduced blood vessel formation. Our results are in agreement with the studies of Fu et al., who reported that RBPMSA and RBPMSC reduced proliferation and migration of breast cancer cells in vitro and in vivo [18].

To further explore the downstream effectors of RBPMSA and RBPMSC in ovarian cancer cells, we performed RNAseq. First, we observed that each RBPMS splice variants regulate its own group of transcripts. Within the RBPMSA downstream transcripts, we identified multiple transcripts of genes associated with chemoresistance, including *NUPR1* and *XAF1* (both increased in our RNAseq). Wen Jiang et al. reported that knockout of *NUPR1* (also known as, *Com-1/p8*) correlated with the increased invasiveness and growth of prostate cancer cells [87]. Overexpression of *NUPR1* reduced the growth of prostate tumors in athymic mice model [87]. *NUPR1* has been shown to interact with transcriptional regulators such as p300, PTIP, estrogen receptor-beta, and Smads [88]. Clack et al. reported that *NUPR1* formed a complex with p53 and p300 in epithelial breast cancer cells [89]. These complexes bound the p21 DNA promoter and transcriptionally upregulated p21 expression [89]. Wen Jiang et al. suggested that in prostate cancer, *NUPR1* acts as a tumor suppressor and facilitator of apoptosis because it was able to trans activates p53 following DNA damage [87]. Interestingly, Jiang et al. reported an association between low levels of *NUPR1* expression with shorter survival in both ERα-positive and ERα-negative breast cancer patients [90]. Together, these observations suggest that RBPMSA could transcriptionally regulate the expression levels of *NUPR1* by interacting with transcriptional regulators. Another possibility is that RBPMSA interacts with the mRNA of *NUPR1* increasing in this way the translation into the *NURP1* protein. These hypothesis needs further investigation.

Increased levels of *LINC01504* and decreased levels of *SNORD99* were also observed in RBPMSA overexpressed cells. Increased levels of *LINC01504* in the non-small cell lung cancers cell lines A549, NCI-H1650, SK-MES-1 and NCI-H226 exposed to cinnamaldehyde promoted the production of cytokine signaling 1 (SOCS1), BTG anti-proliferation factor 2 (BTG2), and Bruton tyrosine kinase (BTK) [32]. Cinnamaldehyde is the main component extracted from cinnamon, which has antiviral and anti-tumor effects in HepG2 hepatocellular carcinoma cell line [91]. *SNORD99*, one of the downregulated transcripts in RBPMSA overexpressed clones, was expressed at a higher level in hepatocellular carcinoma patient tissue samples and in the hepatocellular carcinoma cell lines SK-Hep1 and HCCLM9 [92]. Increased levels of *SNORD99* have been implicated in the regulation of cell proliferation and death balance by promoting cancer cell plasticity [92]. This evidence suggests that RBPMSA could inhibit transcription factors that regulate *SNORD99* expression (i.e., AP-1). Moreover, RBPMSA expression levels could enhance the *LINC01504* levels by promoting its RNA processing.

Overexpression of RBPMSC increased the RNA levels of *DAB2*, *SLFN11*, *FOXD3-AS1*, and *PTGER4*, among others. These transcripts have been endowed with tumor suppressor capabilities and better prognostic patient outcomes [53,93,94]. For example, high levels of *DAB* and *PTGER4*, two of the top upregulated genes in RBPMS clones, act as tumor suppressor genes. Jia et al. reported that in human colorectal cancer, loss of *DAB* increased cellular migration, reduced sensitivity to chemotherapeutic agents, and markedly reduced survival rate [93]. Tseng et al. reported that the phosphorylation of the *DOC-2/DAB2* protein complex inhibited the AP-1 activity [95]. In addition, Murn et al. reported that *PTGER4* knockdown accelerated tumor growth, whereas *PTGER4* overexpression yielded significant protection to B cell lymphoma development through the intrinsic activity between *PTGER4* and *PGE2–EP4* signaling target genes. *PTGER4* expression had an inhibitory effect on the transcriptional activity of the AP-1 components c-Fos and c-Jun [53]. Also, expression of *PTGER4* decreased the expression of IL-2 promoter, which is critically important AP-1 signaling activation [53]. These reports are in agreement with Fu et al. study in where RBPMS splice variants bind to c-Fos and c-Jun and inhibit the binding of the AP-1 complex to its DNA recognition sites [18].

We also observed decreased mRNA levels of *TP63* in RBPMSC overexpressing clones. *TP63* is a critical suppressor of tumorigenesis and metastasis [96]. Sundqvist et al. reported that in the breast cancer cell lines HCC1954, HCC202, MCF10A MI and MII; *TP63* is a AP-1 downstream effector [97]. In the same report, *TP63* strongly potentiates TGFβ induction of AP-1 protein members, in particular c-Fos [97]. Moreover, *TP63* stabilized the interactions between Smads and AP-1, and enhanced the binding of Smads/AP-1 complexed in the chromatin [97]. These reports are in agreement with evidence that RBPMS splice variants interact with Smads and/or c-Jun and c-Fos to regulate AP-1/Smads-dependent genes. Interestingly, Lau et al. reported that *TP63* knowkdown decreased the proliferation of neoplastic stromal cells, throught CDC2 and CDC25C suppression [98]. Also, Seno et al. reported that *TP63* null tymus epithelial cells decreased their proliferative rate as compared with normal cells [99]. These pathways could contribute to the reduced cell proliferation of RBPMS overexpresed clones. However, the mechanism by which RBPMS regulates *TP63* function needs further investigation.

Within the RNA regulated transcripts shared by both, A2780CP20-RBPMSA and A2780CP20-RBPMSC overexpressing clones we identified genes associated with biological processes including ion transportation, lipid biogenesis, collagen remodeling, tumor microenvironment and immune response activity. For example, decreased mRNA levels of *NRP1* were observed in the top 20 RNA transcripts shared between A2780CP20-RBPMSA and A2780CP20-RBPMSC overexpressing clones. Neuropilin-1 (*NRP1*) is a cell surface glycoprotein that has been previously associated with nervous system axonal guidance and as a receptor for the collapsin/semaphorin family of proteins [100]. Soker et al. showed that coexpression of *NRP1* with the kinase insert domain receptor (KDR) increased VEGF, angiogenesis as well as chemotaxis in porcine aortic endothelial cells line PAE [101]. Also, Gagnon et al. reported that inhibition of AP-1 significantly attenuated VEGF-dependent *NRP1* in human umbilical vascular endothelial cells (HUVECs) [70]. These results suggest that RBPMSA and RBPMSC acting together could bind and process RNA transcripts associated with a variety of cellular processes.

Using Kaplan–Meier analysis of publicly available mRNA expression (RNA-Seq data) we further observed that several RNA transcripts differentially abundant in RBPMSA and RBPMSC overexpression clones are significantly associated with survival outcomes in ovarian cancer patients. In particular, we observed that *BST2* (also known as *CD317*), *GBP4,* and *SLC15A3* were associated with OS but not with PFS. Wang et al. observed that high expression of *GBP4* was correlated with good overall survival in cutaneous skin melanoma [30]. *SLC15A3* has been postulated by Song et al. as a prognostic biomarker and target in lung adenocarcinoma [31]. Yi et al. reported that overexpression of *CYP24A1* plays an essential role in enhancing immune activity and inhibiting tumorigenesis [102]. Opposite, *PPP1R1C* has been linked by Liu et al. with the progression and resistance to temozolomide therapy in glioblastoma [51]. Wan et al. identified *FOXD3-AS1* as a cancer-promoting gene in glioma [54]. In addition, Li et al. suggested that downregulation of *COL12A1* has a key role in regulating tumor immune interactions [43]. Therefore, further studies are needed to confirm the biological role of these RBPMS downstream genes and their diagnostic, prognostic and/or therapeutic potential in ovarian cancer.

## 4. Materials and Methods

### 4.1. Cell Lines and Culture Conditions

Human ovarian epithelial endometroid adenocarcinoma cancer cells A2780 and A2780CIS cells were purchased from the European Collection of Cell Cultures (ECACC, Porton Down, Salisbury, UK), and the OVCAR3 cells from the American Type Culture Collection (ATCC, Manassas, VA, USA). The A2780CP20 cells were provided by Dr. Anil K. Sood (MD Anderson Cancer Center, Houston, TX, USA) and have been described elsewhere [9,103,104]. The OVCAR3CIS cells were generated by exposing OVCAR3 to increasing concentrations of cisplatin (CIS; Sigma-Aldrich, St. Louis, MO, USA), as previously described [105]. A2780, A2780CP20, A2780CIS, OVCAR3, and OVCAR3CIS molecular characterization and IC50 values have been previously published [9,20,106]. For propagation A2780, A2780CP20 and A2780CIS were maintained in RPMI-1640 medium (Thermo Scientific, Logan, UT, USA), supplemented with 10% fetal bovine serum (FBS) (Thermo Scientific, Logan, UT, USA) and 0.1% antibiotic/antimycotic solution (Thermo Scientific, Logan, UT, USA). The OVCAR3, and OVCAR3CIS cell lines were maintained and propagated in RPMI-1640 (GE Healthcare Life Sciences, Logan, UT, USA; supplemented with insulin (0.01 mg/mL; Sigma-Aldrich, St. Louis, MO, USA; OVCAR3, OVCAR3CIS) supplemented with 10% FBS, and 0.1% antibiotic/antimycotic solution. All cells were maintained at 37 °C in 5% CO_2_ and 95% air. Cell lines were screened for mycoplasma using the LookOut^®^ Mycoplasma PCR detection kit as described by the manufacturer (Sigma-Aldrich, St. Louis, MO, USA), and authenticated by Promega (Madison, WI, USA) and ATCC using Short Tandem Repeat (STR) analysis. All in vitro experiments were performed with a cell density between 70–85%.

### 4.2. Western Blot Analysis

Cells were detached with Trypsin (0.25%) at 37 °C, washed with Phosphate Buffer Saline (PBS), harvested, and stored at −80 °C until processed. Cells were lysed with ice-cold lysis buffer and incubated on ice for 30 min. Whole cell lysates were centrifuged, supernatants were collected, and protein concentration was determined using Bio-Rad Protein Reagents (Bio-Rad, Hercules, CA, USA). In all cases, protein lysates (50 μg) were separated by SDS-PAGE (12% Acrylamide), blotted onto nitrocellulose membranes, and probed with the appropriate dilution (1:1000) of primary antibody (Sigma, St. Louis, MO, USA; Cat number AV3476). The membranes were rinsed and then incubated with mouse or rabbit IgG horseradish peroxidase (HRP)-linked secondary antibodies (Cell Signaling, 1:5000 dilution). Bound antibodies were detected using enhanced chemiluminescence (GE Healthcare, Logan, UT, USA) followed by autoradiography in a FluorChem^TM^ 8900 (Alpha Innotech Corporation, San Leandro, CA, USA). The intensity of each band was quantified and recorded by Image Lab software (BioRad, Hercules, CA, USA).

### 4.3. RNA Isolation, cDNA Synthesis, and RT-PCR

For the RT-PCR experiment, total RNA was isolated using the GenElute Mammalian Total RNA Miniprep kit from Sigma Aldrich (Cat #RTN350). RNA was converted into complementary DNA (cDNA) with the Sigma-Aldrich Enhanced Avian RT first strand synthesis kit (Cat #STR1-1KT). In brief, total RNA (1 μg), 500 mM dNTP, 2.5 mM random nanomers, and nuclease-free water were mixed for a total volume of 10 mL. The mixture was centrifuged and heated at 70 °C for 10 min. After this period, 1 mL of enhanced avian RT, 2 mL 10 × buffer, 1 mL RNase inhibitor, and nuclease-free water were mixed into each sample. Samples were incubated at 25 °C for 15 min, followed by incubation at 45 °C for 50 min to allow the conversion reaction. The RT-PCR reaction included 12.5 μL Master Mix (JumpStart^TM^ REDTaq → Ready Mix), 1.0 μL forward Primer (10 μM), 1.0 μL Reverse Primer (10 μM), 4.0 μL cDNA, and 6.5 μL RNase free dH_2_O. The PCR cycling conditions were one cycle of initial denaturation of 10 min at 95 °C; 40 cycles of denaturation 15 s at 95 °C; annealing 30 s at 60 °C; and extension 30 s at 72 °C. β-actin was used as an endogenous control. The next primer sequences were used: for RBPMSA forward, 5′-TTCACTGCATGCCCAGATGC-3′, and reverse, 5′-TTCAGCAGAACTGACGGGAC-3′; RBPMSB forward, 5′ CCCAGCTCT GTGAAGGTCAG-3′, and reverse, 5′-GCACTATCAGGAGACGGAGC-3′; RBPMSC forward, 5′-ACACACCTGTCTTTTGTCC ACT-3′, and reverse, 5′-TGCTGGTCTGCAGTAGGTTG-3′; total RBPMS (RBPMST): forward, 5′-CTGTACCCAGCGGAGTTAGC-3′, and reverse, 5′-GTGAAGCGGGA TAGGTGAAA-3′; and β-actin forward, 5′-GAACCCTAAGGCCAAC-3′, and reverse, 5′-TGT CACGCACGATTTCC-3′. The PCR products were separated in 3% tris-borate ethyle nediaminetetraacetic acid (TBE) agarose gel (1% EtBr). Bands were imaged using a gel imager (Gel Doc XR+, Bio Rad).

### 4.4. Stable Transfection for RBPMS exp ression

A2780CP20 cells were seeded in 6 well plates at a concentration of 3.5 × 10^4^ cells/mL and incubated at 37 °C, 5% CO_2_. The next day, pTPC (V123)-RBPMSA (1.0 μg), pTPC (V123)-RBPMSC or an Empty Vector (1.0 μg) pTPC (V123) (transOMIC Technologies, Huntsville, AL) were transfected into the cells using MegaTran 1.0 transfection reagent (1:1 *v*/*v*) (OriGene, Rockville, MD). Twenty-four hours later the culture media was replaced by RPMI-containing puromycin (2.2 mg/mL). The pTPC plasmid contains a puromycin resistance cassette, which will be used for mammalian cell clone selection and maintenance. Cells were growth in media with puromycin until each cell formed an independet colony. Colonies were picked up and allowed to growth as independet clones. RBPMS expression levels in each clone were measured by western blot analysis. These RBPMS overexpressing cells are referred as A2780CP20-RBPMSA and A2780CP20-RBPMSC clones. Also, colonies of A2780CP20 cells transfected with the pTPC-Empty Vector were picked up and growth in independent flasks as individual clones. These A2780CP20 clones are referred as A2780CP20-EV.

### 4.5. Transient Transfection of RBPMSA and RBPMSC in OVCAR3CIS Cells

OVCAR3CIS cells (3.5 × 10^4^ cells/mL) were seeded into 6-well plates. For each well, 1.0 μg of pCMV6-RBPMSA, pCMV6-RBPMSC, or pCMV6 (empty vector) (OriGene Cat #sRC211777, RC200248, and PS100001, respectively) were mixed with MegaTran 1.0 transfection reagent (1:1 *v*/*v*) (OriGene, Rockville, MD, USA) and Opti-MEM medium. The mixture was incubated for 10 min at room temperature and added to the cells. Twenty-four hours later, the medium was replaced with fresh RPMI-1640 (10% FBS, 0.1% antibiotic/antimycotic solution and Kanamycin (25 μg/mL). After 24-h RBPMS expression levels in each clone were measured by Western blots. The RBPMS overexpressing cells were referred as OVCAR3CIS-RBPMSA, OVCAR3CIS-RBPMSC clones and OVCAR3CIS-EV.

### 4.6. Colony Formation, Cell Growth, and Cell Viability Assays

Cell proliferation was assessed by colony formation assays. One-thousand cells of each: A2780CP20-RBPMSA, A2780CP20-RBPMSC or A2780CP20-Empty Vector (A2780CP20-EV) clones were seeded in 10-cm Petri dishes (2.0 × 10^4^ cells/mL). Seven to ten days later, colonies were stained with 0.5% crystal violet in methanol. Colonies more than 50 cells were counted in five random fields (10×) using the Nikon Eclipse TS100 microscope (Nikon, Minato, Tokyo, Japan). The percentage of colonies was calculated relative to the number of colonies in the A2780CP20-EV plate, which was considered as 100%.

For cell viability, cells (3.5 × 10^4^ cells/mL) were seeded into 96-well plates and 24 h later exposed to different concentrations of cisplatin (0.1 µg/mL, 1.0 µg/mL, 10 µg/mL, 25 µg/mL, 50 µg/mL, 100 µg/mL) and incubated for 72 h (Sigma-Aldrich, St. Louis, MO, USA). After this period of time, the medium was removed and 100 µL of Alamar blue (Invitrogen) dye was added. The optical density (OD) values were obtained spectrophotometrically in a plate reader (BioRad, Hercules, CA, USA) after a maximum of 4 h of dye incubation. In all cases, percentages of cell viability were obtained after blank OD subtraction, taking the untreated cells values as a normalization control. For the cell growth curve cells (2.0 × 10^4^ cells/mL) were seed in a 10-cm Petri dishes and incubate for 24 h at 37 °C. Cells were detached with Trypsin (0.25%) at 37 °C, staining with 0.5% trypan blue solution and counted in triplicates at 24 h interval for 96 h after plating using a hemocytometer.

### 4.7. Migration and Invasion Assays

Cell migration was measured with the wound healing assay and cell invasion by the matrigel transwell method, as previously described [17,107]. For invasion assay, cells (3.5 × 10^4^ cells/mL) were seeded into 6-well plates. The next day, Matrigel (BD Biosciences, San Jose, CA, USA) in serum-free medium was added onto the upper chambers of 24 transwell plates and incubated at 37 °C for polymerization. Clones and controls cells were collected and resuspended in serum-free medium and re-seeded onto the Matrigel-coated chamber. Medium containing 10% FBS was added to the lower part of the wells and plates were incubated for 48 h at 37 °C. Then, the medium was removed, and cells that had invaded through the Matrigel were fixed and stained using the Protocol Hema 3 Stain Set (Fisher Scientific, Kalamazoo, MI, USA). The invading cells were counted at 20× using an Olympus 1 × 71 microscope equipped with a digital camera (Olympus DP26). The cell invasion percentages were calculated by assuming the A2780CP20-EV values in terms of 100% cell invasion. For the wound healing assay cells were seeded into 6-well plates and scraped with the 200 µL pipette tips. The plates were washed with PBS to remove detached cells and then incubated with the proper growth media for 24 h. Cell migration was observed under a phase contrast microscope at 20× magnification at 0, 12 and 24 h post-induction of injury. Migrated cells in the clean area in each of five random fields were measured and quantified using Nikon Eclipse Ts2R microscope with the Nikon DS-Qi2 camera and subsequently analyzed with the NIS-Element Microscope Software.

### 4.8. Mice Experiments

Female BALB/c nude mice (4–6 weeks of age) were purchased from Taconic Biosciences (Rensselaer, NY, USA). Cells (2.0 × 10^6^ cells/200 μL in PBS/Matrigel mixture) were subcutaneously injected into the right dorsal flank. The tumor growth was monitored with a Vernier caliper three times per week. Tumor volumes were calculated using the following formula: volume = (L × W × H) × 0.5, where L is the length (longest diameter), W is the weight (thickness), and H is the height (shorter diameter). The size and weight of the tumors as well as number of nodules was recorded. Animal handling and research protocols were approved by the Institutional Animal Care and Use Committee (IACUC) of the University of Puerto Rico, Medical Sciences Campus on 25 January 2022 (protocol number: A870110).

### 4.9. Immunohistochemistry

Pieces of tumors collected from mice experiments were fixed on paraffin blocks and sectioned (5 μm thick). The slides were then deparaffinized, re-hydrated, and then immersed in distilled water with 3% hydrogen peroxidase to suppress endogenous peroxidase activity. Antigen retrieval of tissue sections was performed by microwave treatment in antigen unmasking solution (Vector Laboratories, Inc, Burlingame, CA, USA) for 15 min. Sections were incubated with RBPMS antibody, proliferation antibody Ki67 or anti-VEGF antibody CD31 (Abcam, Cambridge, MA, USA) at a dilution of 1:100, 1:500 and 1:100 respectively; in Dako antibody diluent (Dako North America Inc, Carpinteria, CA, USA) overnight at 4 °C. Subsequently, the Envision peroxidase-labeled polymer (goat anti-mouse; Dako North America Inc, Carpinteria, CA, USA) was applied to the sections and signals were developed with diaminobenzidine (DAB) chromogen. Three slides per mice were analyzed. Images from five microscopic fields per slide was taken using an Olympus 1 × 71 microscope equipped with a digital camera (Olympus DP26). The immunoreactivity was estimated and compared using Student’s *t*-test for comparing two groups and by ANOVA for multiple group comparisons. *p*-values of <0.05 were considered statistically significant.

### 4.10. Senescence Associated β-Galactosidase Activity Assays

We performed the senescence assays using the beta-galactosidase (β-Gal) Detection Kit from Abcam (Cat #AB176721). Cells were collected, lysed and diluted to a final concentration of 1 µg/mL. The samples were incubated for four hours with FDG. Next, stop buffer was added, and fluorescence was quantified using the Thermo Scientific Varioskan Flash spectral reader machine at 490 nm excitation and 525 nm emission. β-Gal levels were calculated with respect to the standard curve prepared for each experiment. To assess the senescence associated β-galactosidase staining, we seeded 30,000 cells of each cell type (A2780-CP20, A2780CP20-EV, A2780CP20-RBPMSA (clones 7 and 8) and A2780CP20-RBPMSC (clones 3.3 and 3.10) per well in a 6-well plate. Twenty-four hours later, the β-galactosidase staining was assessed using a senescence detection kit (Ab65351, Abcam, Cambridge, MA, USA) following the manufacturer’s recommendations. Cell images were taken at 20× on an Olympus 1 × 71 microscope.

### 4.11. RNA Sequencing Library Preparation, Data Processing, and Statistics

For the RNA sequencing library preparation, RNA was extracted from A2780CP20-EV, A2780CP20-RBPMSA, and A2780CP20-RBPMSC using the Qiagen RNeasy Kit (Cat #74004). Agilent RNA TapeStation, and 1 µg of high-quality RNA was used for polyA mRNA enrichment (RIN > 9.7) to verified the RNA quality. Next, according to manufacturer protocol NEBNext polyA mRNA magnetic isolation module (NEB #E7490) was used for purificate the polyA mRNAs. Them the mRNA samples isolated in the previous step were fragmented into ~200 bp and purified for the library preparation. Following the manufacturer’s protocol cDNA, ligation and DNA amplification were done. Using NEBnext sample purification beads, and Agilent HS-DNA Tapestation analysis the resulted library was purified and suggested to a quality control step. In a final concentration of 5 nM the samples were multiplexed and sequenced on the IlluminaNovaseq platform. For the RNA sequenceing analysis, the reads were adapter and trimmed using TrimGalore-0.6.0. Contaminating reads from ribosomal RNA was removed and transfer to RNA using Bowtie2 (version 2.2.9) [108,109]. The trimmed and contamination filtered reads were mapped to the hg38 genome (GENCODE Release 31) using STAR aligner version 2.5.2a, and a count matrix was obtained using the “Gene Counts” option [110]. The differential gene expression analysis were done using the DESeq2 (version 1.28.1) package in R version 4.0.1 [111]. Ensembl IDs were converted to gene symbols and names using the org.Hs.eg.db package (version 3.11.4). Significance was set at an FDR-adjusted *p*-value < 0.05 and fold change ≥ |2.0|.

### 4.12. Validation of the RNAseq Results by SYBR-GREEN Based qRT-PCR

To validate de differentially regulated genes obtained by RNAseq, we purchased a custom 384-well PCR plate (Bio-Rad (Hercules, CA, USA). The plate contained specific pre-designed forward and reverse primers to each gene. Following the manufacturer’s instructions total RNA from A2780CP20-EV, A2780CP20-RBPMSA and A2780CP20-RBPMSC clones were isolated using the GenElute Mammalian Total RNA Mini Kit (Millipore-Sigma, St. Louis, MO, USA). Reverse transcription of the total RNA was performed using the iScript Reverse Transcription Supermix for RT-qPCR (Bio-Rad, Hercules, CA, USA, Cat #1708841). SsoAdvanced™ Universal SYBR^®^ Green Supermix (Bio-Rad, Hercules, CA, USA, Cat #1725271) and CFX384 Touch Real-Time PCR detection system was used for the SYBRI Green-based qPCR assay. Instrument’s internal software calculate the fold-changes and cycle threshold (Ct) values relative to A2780CP20-EV samples and normalized to β-actin. Others controls such as gDNA, PCR reaction, RT reaction, and RNA quality inlcuded in the PCR plate were added in the analysis as controls.

### 4.13. Clustering and Network Analysis

To determine the functional networks and pathways associated with the differentially abundant transcripts identified by the RNAseq, Ingenuity Pathway Analysis (IPA) (Ingenuity Systems, Qiagen, Redwood City, CA, USA) was performed. A a fold change ≥ |2.0| and *p*-value ≤ 0.05 was the cutoff for considering significant a gene or proteins in the IPA CORE analysis; the human was considered as the model organism for annotations [112]. Using Metascape a Gene Annotation & Analysis Resource we performed the Network and canonical pathway enrichment analyses, filtering for all tissues, cell lines, and human species [113].

### 4.14. Kaplan-Meyer (KM) Survival Analysis

Kaplan Meyer (KM) plots analysis was performed using available gene chip and RNA-Seq datasets in the publicity KM plotter database (www.kmplot.com) (accesed on 15 July 2022) [114]. For each gene symbol, a probe ID was selected, and the ovarian cancer patients were categorized into high or low expression groups based on the RNA expression median values of the dataset. When gene had multiple probes, the best probe was selected. All available datasets were used for survival analysis. KM survival plots for OS and PFS were generated with their respective hazard ratios (HRs), confidence intervals (CIs), and *p*-values (log-rank). *p*-values < 0.05 were considered statistically significant.

### 4.15. Statistical Analysis

All experiments were performed in triplicates and analyzed using GraphPad Prism 7 (GraphPad Software, La Jolla, CA, USA). Statistical differences were determined using a 2-tailed, unpaired Student’s *t*-test and one-way and two-way ANOVA tests were performed as per the requirement of the analysis * *p* ≤ 0.05, ** *p* ≤ 0.01, *** *p* ≤ 0.001, **** *p* ≤ 0.0001. *p*-value of less than 0.05 was considered statistically significant.

## 5. Conclusions

In summary, inceased expression of RBPMSA and RBPMSC variants in ovarian cancer cells reduced cell proliferation, invasion, and migration of these cells. However, only RBPMSA was associated with the cisplatin sensitivity of ovarian cancer cells. RBPMSA and RBPMSC control the expression of RNA transcripts associated with the remodeling of the tumor microenvironment, cell proliferation, cell survival, and cell integrity, among others. These findings highlight the important role of RBPMS splice variants in the regulation of gene expression in health and disease.

## Figures and Tables

**Table 1:** Top 20 differentially expressed RNA transcripts in A2780CP20-RBPMSA vs. A2780CP20-EV clones.

Symbol	Gene Name	Fold Change	Biological Role	Reference
IFI44	Interferon induced protein 44	9.66541828	Plays a role in the immune response during autoimmune diseases.	[29]
XAF1	XIAP Associated Factor 1	8.297767889	A putative tumor suppressor candidate that junction to several pathways leading to apoptosis.	[21]
GBP4	Guanylate Binding Protein 4	6.931543382	Involved in the host-defense mechanisms response against cellular pathogens and tumorigenesis.	[30]
SLC15A3	Solute Carrier Family 15 Member 3	6.865730827	Transporting histidine, peptides and peptidomimetics from inside the lysosome to cytosol.	[31]
RBPMS	RNA Binding Protein	6.758908087	Regulate the RNA transport, stability and localization.	[17]
LINC01504	Long Intergenic Non-Protein Coding RNA 1504	6.554246988	A lncRNA which has a role on the suppression of malignant phenotypes of lung cancer.	[32]
NUPR1	Nuclear Protein 1, Transcriptional Regulator	6.087442834	Upregulation of this protein is associated with malignant characteristics of cancer as well as with chemoresistance.	[22]
BST2	Bone Marrow Stromal Cell Antigen 2	5.971957997	Lipid raft-associated type II transmembrane glycoprotein which mediates various facets of cancer progression and metastasis	[33]
FGF21	Fibroblast Growth Factor 21	5.930365363	Member of the FGF family which possess broad mitogenic and cell survival activities.	[34]
HSH2D	Hematopoietic SH2 Domain Containing	5.864666169	Play a role in various cellular functions such as apoptosis, membrane-associated intracellular trafficking and the biogenesis of lipid and collagen remodeling.	[35]
S100A2	S100 Calcium Binding Protein A2	−2.477696881	Plays a role in metastasis process by transforming growth factor-β (TGF-β) mediated cancer cell invasion and migration.	[36]
KCNH4	Potassium Voltage-Gated Channel Subfamily H Member 4	−2.510279699	Transport positively charged potassium atoms between neighboring cells. KCNH4 plays a key role in the ability of cells to generate and transmit electrical signals.	[37]
SNORD99	Small Nucleolar RNA, C/D Box 99	−2.521724113	Related with diverse cellular functions such as regulation of T cell proliferation and death balance to promoting cancer cell plasticity.	[38]
LRRC8D-DT	LRRC8D Divergent Transcript	−3.051305443	Plays important pharmacological and physiological roles in supporting the transport of anti-cancer drugs and of the organic osmolyte taurine.	[39]
TXK	TXK Tyrosine Kinase	−3.120303742	Play important roles in the immune response and pathway signaling mediator	[40]
SGCZ	Sarcoglycan Zeta	−4.110780038	Part of the sarcoglycan complex which have a structural role in connecting cytoskeletal proteins with the extracellular matrix.	[41]
HIST1H2BH	H2B Clustered Histone 9	−4.323395136	Responsible for the nucleosome structure of the chromosomal fiber in eukaryotes. Low levels of HIST1H2BEH caused decreased proliferation in breast cancer cell lines.	[42]
COL12A1	Collagen Type XII Alpha 1 Chain	−4.332051747	Found in several cancer types and could be involved in tumor progression.	[43]
PREX2	Phosphatidylinositol-3,4,5-Trisphosphate Dependent Rac Exchange Factor 2	−4.381347741	Implicated in the inhibition of phosphatase and tensin homolog (PTEN). Overexpression significantly increases the proliferation, invasion, and migration of pancreatic cancer.	[44]
CCL2	C-C Motif Chemokine Ligand 2	−4.644149886	Strongest chemoattractant synthesized and secreted mainly by monocytic cells.	[45]

**Table 2:** Top 13 differentially expressed RNA transcripts in A2780CP20-RBPMSC vs. A2780CP20-EV clones.

Symbol	Gene Name	Fold Change	Biological Role	Reference
DAB2	DAB Adaptor Protein 2	7.15380118	Multi-function signaling molecule which catalytic enzyme activity suggest that it is an adaptor molecule involved in multiple receptor-mediated signalling pathways that plays a pivotal role in the cellular homeostasis.	[46]
CALB2	Calbindin 2	6.574845254	Important mediator of 5-FU-induced cell death and specific marker for the diagnosis of malignant mesothelioma.	[47]
CTNND2	Catenin Delta 2	6.484328261	Recognized to be a biomarker for cancers, overexpressed in various types of cancers, including prostate, breast, lung and ovarian cancer.	[48]
CYP24A1	Cytochrome P450 Family 24 Subfamily A Member 1	6.041287981	Member of the cytochrome P450 superfamily of enzymes which catalyze many reactions involved in drug metabolism and synthesis of cholesterol, steroids and other lipids.	[49]
FAR2P2	Fatty Acyl-CoA Reductase 2 Pseudogene 2	5.29742507	Catalyzes the reduction in saturated but not unsaturated C16 or C18 fatty acyl-CoA to fatty alcohols.	[50]
RBPMS	RNA Binding Protein	4.920050075	Regulate the RNA transport, stability and localization.	[17]
PPP1R1C	Protein Phosphatase 1 Regulatory Inhibitor Subunit 1C	4.253043369	Major serine/threonine phosphatase that regulates a variety of cellular functions and themselves regulated by phosphorylation.	[51]
SLFN11	Schlafen Family Member 11	3.827804248	DNA/RNA helicase that is recruited during stressed replication fork and irreversibly triggers replication block and cell death.	[52]
PTGER4	Prostaglandin E Receptor 4	3.770525307	Member of the G-protein coupled receptor family which bind and mediate cellular responses to PGE2 and other prostanoids.	[53]
FOXD3-AS1	FOXD3 Antisense RNA 1	3.654548595	Is abnormally expressed in many disease types. Reports suggest that FOXD3-AS1 is highly expressed in different cancer types promoting migration and invasion capacity.	[54]
TP63	Tumor Protein P63	−2.226163472	Functions as a transcription factor interacting with other proteins to turn different genes on and off at different times.	[23]
DTNA	Dystrobrevin Alpha	−2.582128781	Belongs to the dystrobrevin subfamily of the dystrophin family. Reports suggest that DTNA binds and activates STAT3 to induce TGFβ1 expression and repress P53 expression.	[55]
SCN3A	Sodium Voltage-Gated Channel Alpha Subunit 3	−4.437260362	Is a transmembrane glycoprotein responsible for the generation and propagation of action potentials in neurons and muscle.	[56]

**Table 3:** Top 20 RNA transcripts shared by A2780CP20-RBPMSA and A2780CP20-RBPMSC clones.

Symbol	Gene Name	Fold Change	Biological Role	Reference
FAR2P2	Fatty Acyl-CoA Reductase 2 Pseudogene 2	5.29742507	Acts as guanine nucleotide exchange factor that activates RAC1. Also, plays a role in the response to class 3 semaphorins and remodeling of the actin cytoskeleton.	[50]
RBPMS	RNA Binding Protein	4.920050075	Regulate the RNA transport, stability and localization.	[17]
ANKRD33B	Ankyrin Repeat Domain 33B	4.556503793	Involved in negative regulation of transcription by RNA polymerase II and negative regulation of transcription regulatory region DNA binding activity.	[57]
PPP1R1C	Protein Phosphatase 1 Regulatory Inhibitor Subunit 1C	4.253043369	Major serine/threonine phosphatase that regulates a variety of cellular functions and themselves regulated by phosphorylation.	[51]
FGF12	Fibroblast Growth Factor 12	3.920423579	Involved in a broad mitogenic and cell survival activities, including embryonic development, cell growth, morphogenesis, tissue repair, tumor growth, and invasion.	[58]
GABRA2	Gamma-Aminobutyric Acid Type A Receptor Subunit Alpha2	3.844344607	Component of the heteropentameric receptor for GABA, the major inhibitory neurotransmitter in the brain.	[59]
FOXD3-AS1	FOXD3 Antisense RNA 1	3.654548595	Is abnormally expressed in many disease types. Reports suggest that FOXD3-AS1 is highly expressed in different cancer types promoting migration and invasion capacity.	[60]
NFATC1	Nuclear Factor of Activated T Cells 1	3.620469318	Family of proteins that play a central role in inducible gene transcription during immune response.	[61]
ROBO2	Roundabout Guidance Receptor 2	3.448549593	Transmembrane receptor for the slit homolog 2 protein that play a function in axon guidance and cell migration.	[62]
CDH6	Cadherin 6	3.421265843	Membrane glycoprotein that mediates homophilic cell-cell adhesion and play critical roles in cell differentiation and morphogenesis.	[63]
HOXD8	Homeobox D8	−2.593778164	Gene belongs to the homeobox family of genes which play an important role in morphogenesis in all multicellular organisms.	[64]
MYL7	Myosin Light Chain 7	−2.677248207	Part of the family motor proteins that have ATPase enzyme activity, actin binding and potential for kinetic energy transduction.	[65]
SSUH2	Ssu-2 Homolog	−2.71336991	Gene that encodes a protein tyrosine phosphatase that plays a key role in the regulation of actin filaments.	[66]
HOXD9	Homeobox D9	−2.800133712	Transcription factor which is part of a developmental regulatory system providing cells the specific positional identities on the anterior-posterior axis.	[67]
DAPK1	Death-Associated Protein Kinase 1	−3.221475672	Mediator of gamma-interferon involved in multiple cellular signaling pathways that trigger cell survival, apoptosis, and autophagy.	[68]
SNTG1	Syntrophin Gamma 1	−3.228723507	Cytoplasmic peripheral membrane proteins that contain 2 pleckstrin domains.	[69]
NRP1	Neuropilin 1	−3.454159744	Cell membrane receptor involved in the development of cardiovascular system, angiogenesis, certain neuronal circuits and organogenesis in nervous system.	[70]
ERICH3	Glutamate Rich 3	−3.951576843	Interacts with proteins function in vesicle biogenesis and may play a significant role in vesicular function in serotonergic and other neuronal cell types.	[71]
JAG1	Jagged Canonical Notch Ligand 1	−6.91254142	Ligand for multiple Notch receptors involved in the mediation of Notch signaling, cell-fate decisions during and cardiovascular development.	[72]
TRBV12-4	T Cell Receptor Beta Variable 12-4	−6.91254142	Antigen specific receptor which are essential to the immune response and are present on the cell surface of T lymphocytes	[73]

## Data Availability

Not applicable.

## Supplementary Materials

The following supporting information can be downloaded at: https://www.mdpi.com/article/10.3390/ijms232314742/s1.
