# Supplementary material for: Increased Expression of the RBPMS Splice Variants Inhibits Cell Proliferation in Ovarian Cancer Cells
Source: Int J Mol Sci. 2022 Nov 25;23(23):14742. doi: 10.3390/ijms232314742 (PMC9738375; doi:10.3390/ijms232314742)
Supplement: Supplementary file 1 [file ijms-23-14742-s001.zip › Supplementary Materials.pdf]

Supplementary Materials

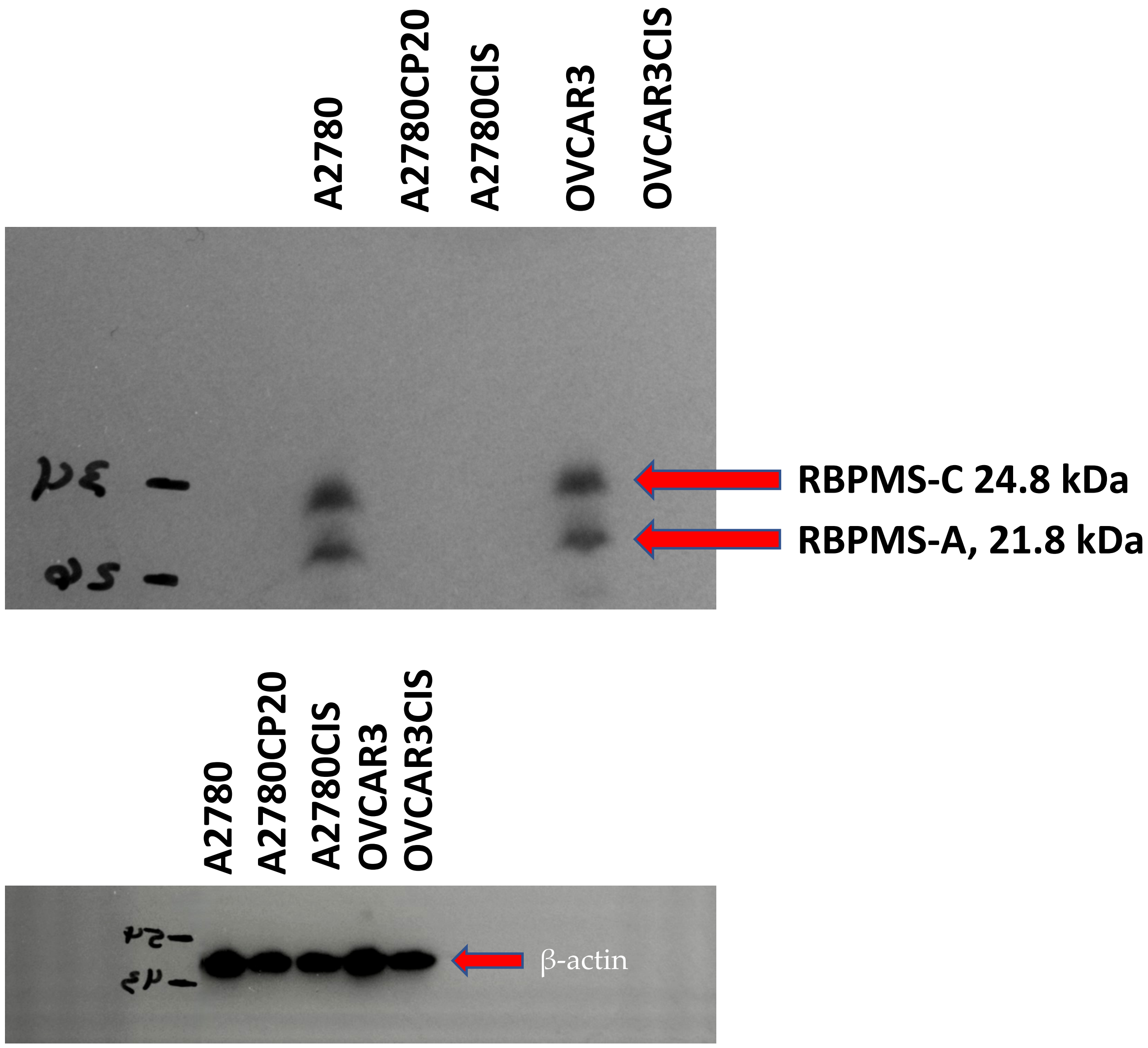

Supplementary Figure S1. Original western blot images showed in Figure 1A.

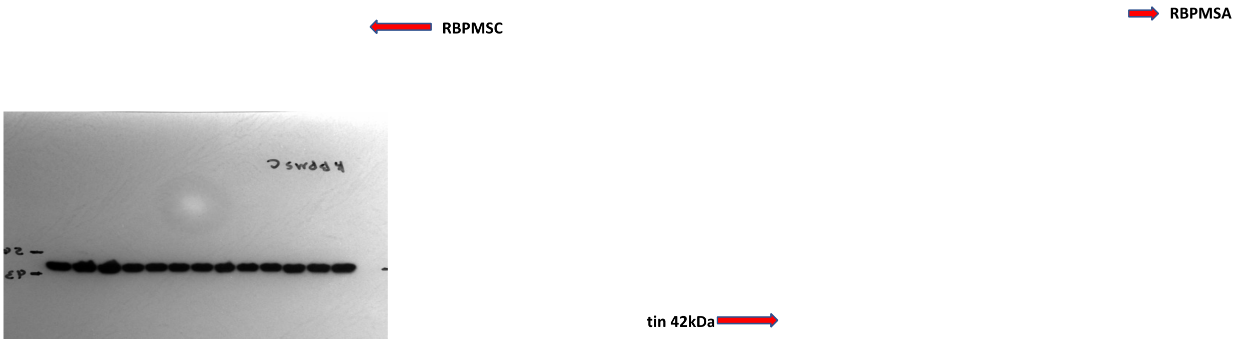

Supplementary Figure S2. Original western blot images of the Western blots showed in Figure 1H and 1I.

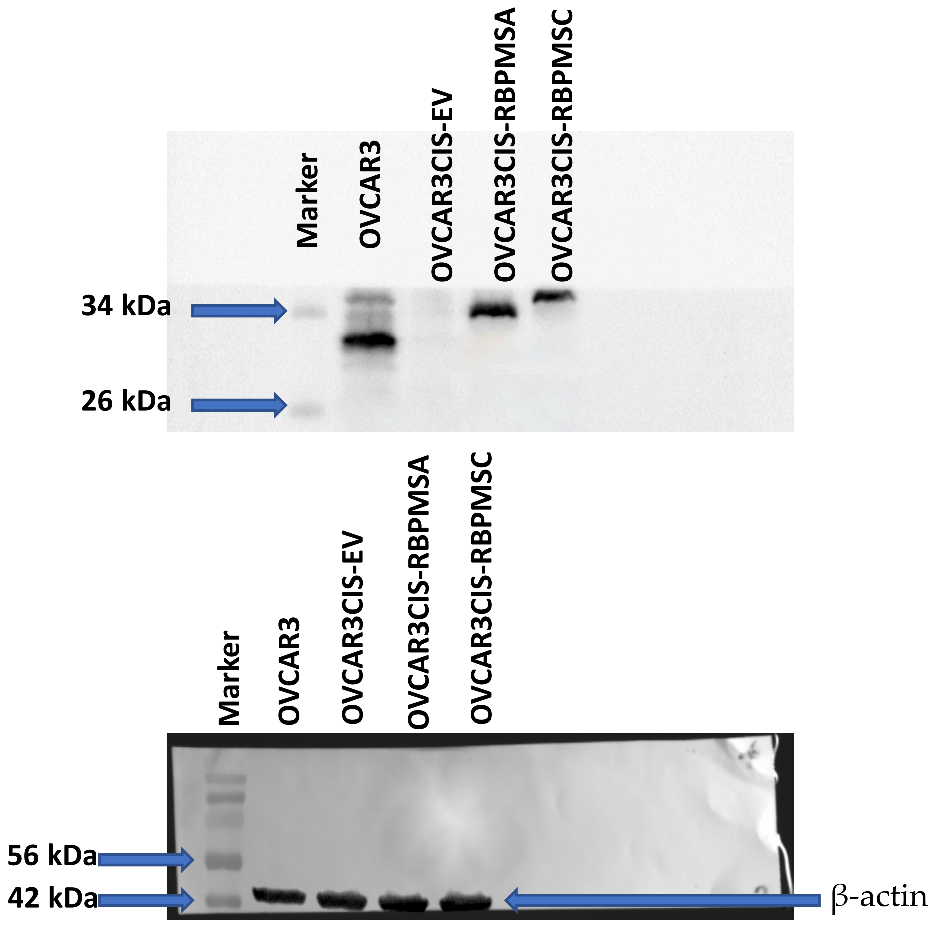

Supplementary Figure S3a. Original western blot images of the Western blots showed in Figure 2E.

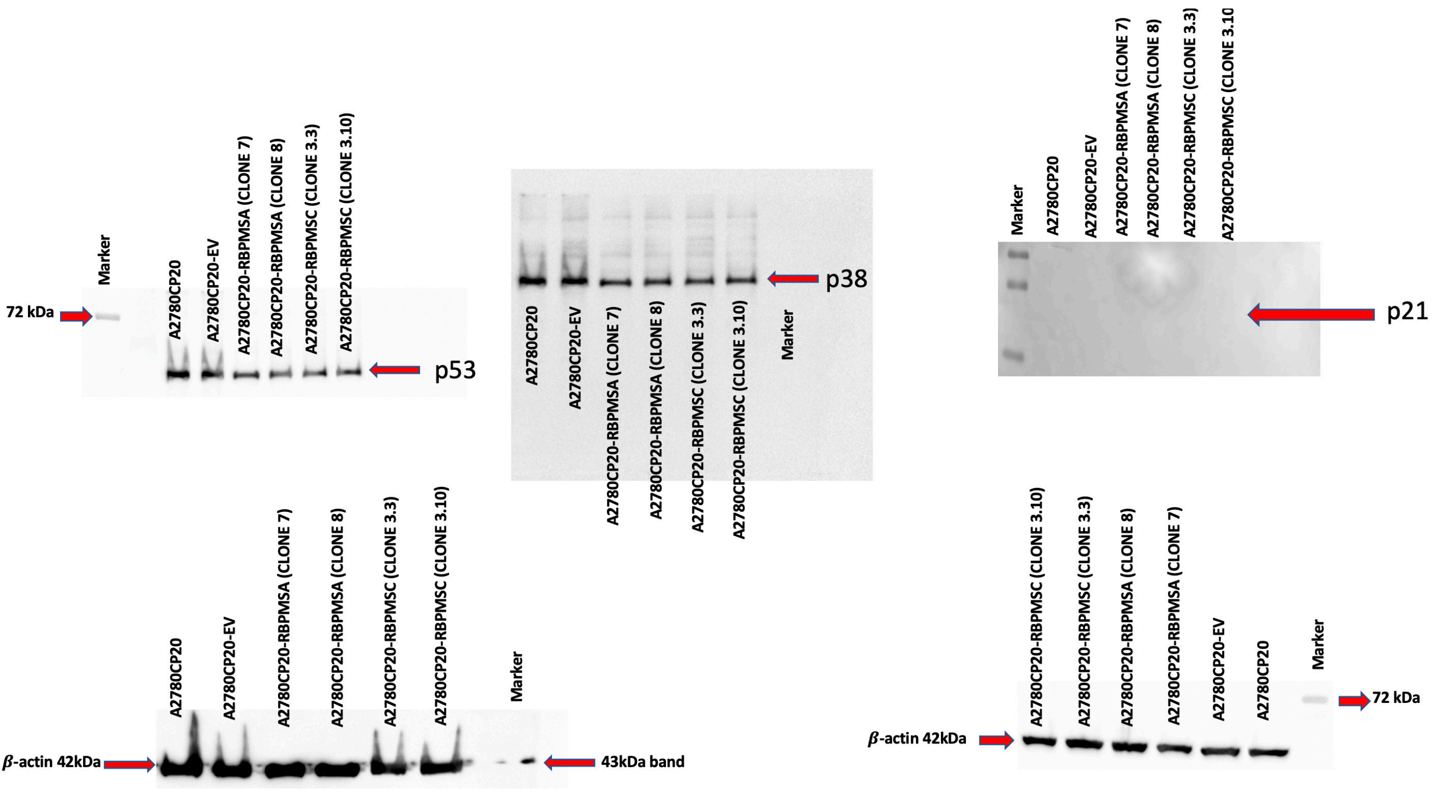

Supplementary Figure S3b. Original western blot images of the p53, p21, p38 Western blots showed in Figure 3D.

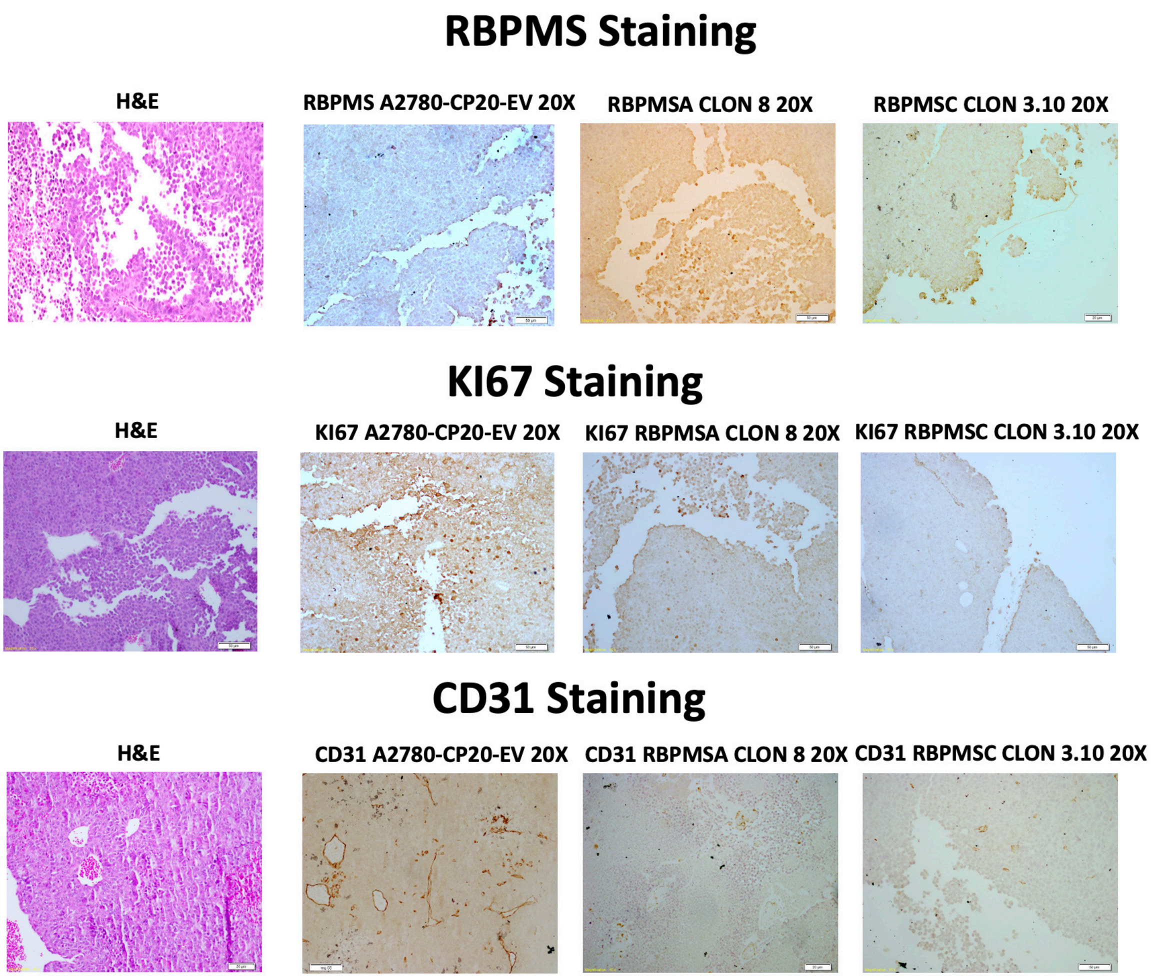

Supplementary Figure S4. Representative images of IHC analysis of RBPMS expression (anti-RBPMS), proliferation (KI-67), and blood vessels formation (CD31). Microscopy images were taken at 20X.

Supplementary Table S3. Relative expression values of the differentially expressed RNA transcripts in A2780CP20-RBPMSA vs. A2780CP20-EV clones.

RBPMS-A Transcripts Validation Expression		
Gene ID	RT-qPCR FC	RNA-Seq FC
**IF144**	**9.666**	**9.665**
**XAF1**	**8.298**	**8.297**
**GBP4**	**6.932**	**6.931**
**NUPR1**	**6.087**	**6.087**
**BST2**	**5.972**	**5.971**
**HSH2D**	**5.865**	**5.864**
**COL12A1**	**-4.332**	**-4.332**
**LLRC8D-DT**	**-3.050**	**-3.058**
SLC15A3	-2.273	6.865
**RBPMS**	**6.499**	**6.758**

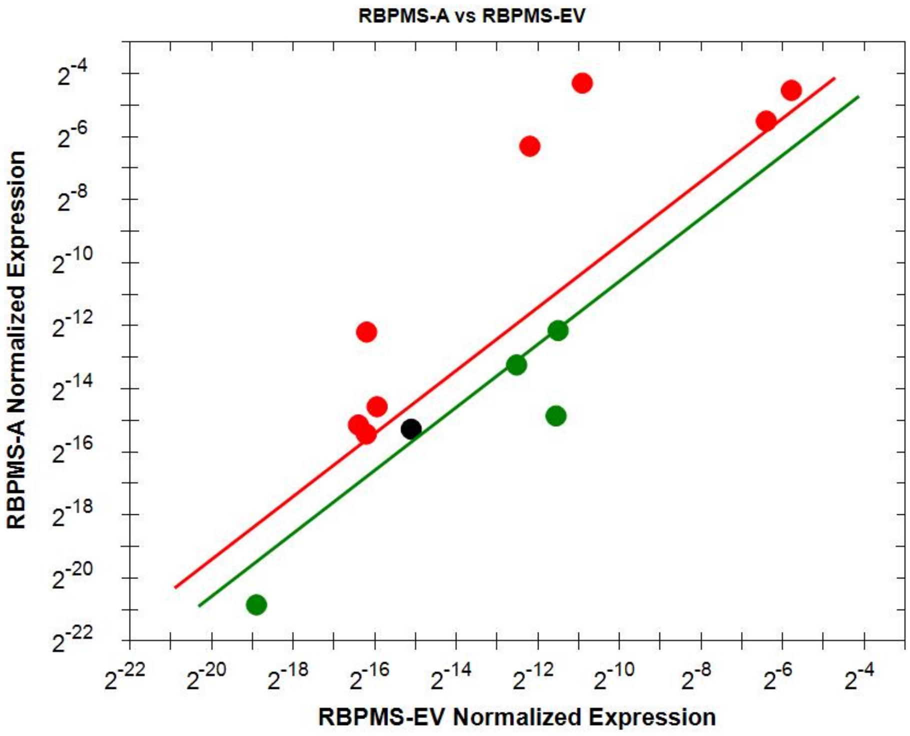

Supplementary Figure S5. Validation of the 10 differentially abundant transcripts by RT−qPCR in A2780CP20-RBPMSA clones. Expression values were calculated relative to A2780CP20-EV clones. Green dots represent downregulated transcripts and red dots represented upregulated genes. Selected threshold for significant fold changes values are represented by the green and red lines in the image.

Supplementary Table S4. Relative expression values of the differentially expressed RNA transcripts in A2780CP20-RBPMSC vs. A2780CP20-EV clones.

RBPMS-C Transcripts Validation Expression		
Gene ID	RT-qPCR FC	RNA-Seq FC
**DAB2**	**7.154**	**7.15**
**CALB2**	**6.575**	**6.57**
**CYP24A1**	**6.041**	**6.041**
**SLFN11**	**3.828**	**3.827**
**PTGERR4**	**3.771**	**3.770**
TP63	2.861	-2.226
DTNA	2.783	-2.582
SCN3A	2.775	-4.437

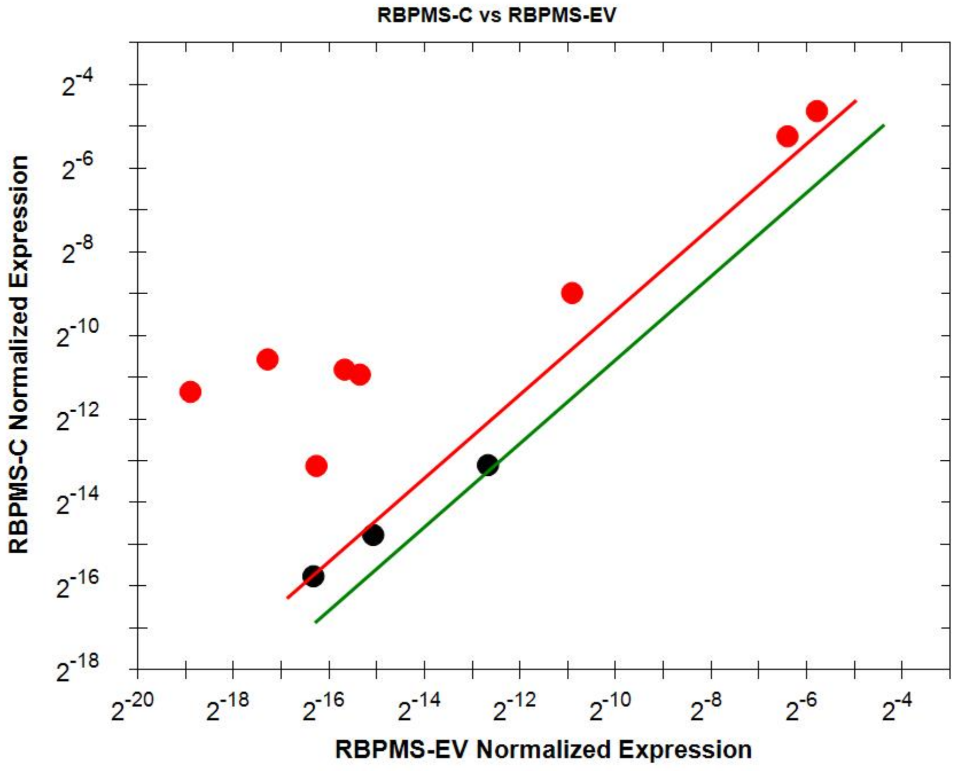

Supplementary Figure S6. Validation of the 8 differentially abundant transcripts by RT−qPCR in A2780CP20-RBPMSC clones. Expression values were calculated relative to A2780CP20-EV clones. Green dots represent downregulated transcripts and the red dots represented upregulated genes. Selected threshold for significant fold changes values are represented by the green and red lines in the image.

Supplementary Table S5. Top canonical pathways generated with the unique and common deregulated RNAs in A2780CP20-RBPMSA and A2780CP20-RBPMSC.

Canonical Pathways for A2780CP20-RBPMSA			
Ingenuity Canonical Pathways	***log10 (p-Value)***	***Number of Genes***	***Genes***
Cancer, Cardiovascular System Development and Function Organismal Development	1.24 x 10^{-03}	31	ADAM17, ANXA3, ARHGDIB, ATP5MC1, AXL, C3, CDH2, CLDN7, CXCL8, DDR2, E2F3, EGFR, GAS6, IFI44, IRF1, ITGB1, LGALS3, LOXL2, MMP1, MMP2, MMP9, MRPL36, NCOA7, PHGDH, PRSS8, PTGS2, SNAI1, SPINT1, TIMP2, TWIST1, UBE2L2
Cell Cycle, Cellular Development, Cellular Growth, and Proliferation	4.30 x 10^{-03}	25	ARID1A, BMI1, BTG2, CAMKK2, CCND1, CCNE1, CCNE2, CDK2, CDK4, CDK6, CDKN1A, CDKN18, COL6A1, CYFIP2, DHRS3, GDF15, GGT7, HDAC5, ITGB4, PTPRR, RB1, SALL2, SIN3A, TERT, TP53
Antimicrobial Response, Inflammatory Response, and Organismal Injury and Abnormality	3.54 x 10^{-02}	35	BBC3, CASP1, CCL26, CD70, CD83, CHIT1, COLEC12, DDX58, DLX4, DUSP4, EPAS1, FGF2, HLA-A, ICAM1, IFI27, IFI35, IFIH1, IFIT1, IFIT2, IL18, IL23A, IRAK2, IRF7, ISG20, MAVS, NOS2, PMAIP1, RELB, SERPING1, STING1, THBS1, TNFRSF11B, TP73, TXNIP, VEGFA
Cell Cycle, Gene expression, Cellular Growth, and Proliferation	4.64 x 10^{-02}	18	ATF3, BRCA1, CCNG2, CLSPN, CPM, ETV6, FOS, FOXL2, FOXO3, GNRHR, HERC2, JUN, JUNB, MAFF, NODAL, RAF1, SMAD4, SOX4
Cellular Function and Maintenance	4.64 x 10^{-02}	4	AR, MAP2K6, MYOF, PGK1
Canonical Pathways for A2780CP20-RBPMSC			
Ingenuity Canonical Pathways	***log10 (p-Value)***	***Number of Genes***	***Genes***
Cardiovascular System Development and Function, Cell to Cell Signaling and Interaction, Cellular Movement	6.64 x 10^{-03}	2	CCN2, CLDN7
Organ Morphology, Reproductive System Development and Function, Tissue Development	4.56 x 10^{-02}	3	CAV1, EGFR, ITGA2
Antimicrobial Response, Cell Cycle and Survival	3.15 x 10^{-02}	2	BIRC3, PPKAR2B
Cancer, Cellular Movement, Organismal Injury and Abnormality	2.68 x 10^{-03}	2	BDNF, TUG1
Cell Morphology, Cell to Cell Signaling and Interaction, Cellular Development	2.68 x 10^{-03}	2	AKT1, TP63
**Common** *Canonical Pathways Between RBPMSA and RBPMSC*			
Ingenuity Canonical Pathways	***log10 (p-Value)***	***Number of Genes***	***Genes***
Cancer, Cardiovascular Disease Hematological System Development and Function	4.67 x 10^{-03}	2	CLDN7, F3
Cell to Cell Signaling and Interaction, Cellular Development, Cellular growth, and Proliferation	4.38 x 10^{-03}	2	FOS, MECOM
Cancer, Cellular Movement, Organismal Injury and Abnormality	3.44 x 10^{-02}	2	BDNF, TUG1
Cell Death and Survival, Molecular Transport, Protein Trafficking	2.82 x 10^{-02}	2	ANKRD1, DDIT3
Cancer, Cell to Cell Signaling and Interaction, Dermatological Disease and Conditions	1.73 x 10^{-03}	2	LRP1, PTPRR
